# The Development and Validation of the Epistemic Vice Scale

**DOI:** 10.1007/s13164-021-00562-5

**Published:** 2021-06-25

**Authors:** Marco Meyer, Mark Alfano, Boudewijn de Bruin

**Affiliations:** 1 Department of Philosophy, University of Hamburg, Hamburg, Germany; 2 Faculties of Philosophy and of Economics and Business, University of Groningen, Groningen, The Netherlands; 3 Faculty of Philosophy, Macquarie University, Sydney, Australia

## Abstract

This paper presents two studies on the development and validation of a ten-item scale of epistemic vice and the relationship between epistemic vice and misinformation and fake news. Epistemic vices have been defined as character traits that interfere with acquiring, maintaining, and transmitting knowledge. Examples of epistemic vice are gullibility and indifference to knowledge. It has been hypothesized that epistemically vicious people are especially susceptible to misinformation and conspiracy theories. We conducted one exploratory and one confirmatory observational survey study on Amazon Mechanical Turk among people living in the United States (total *N* = 1737). We show that two psychological traits underlie the range of epistemic vices that we investigated: indifference to truth and rigidity. Indifference manifests itself in a lack of motivation to find the truth. Rigidity manifests itself in being insensitive to evidence. We develop a scale to measure epistemic vice with the subscales indifference and rigidity. The Epistemic Vice Scale is internally consistent; has good convergent, divergent, and discriminant validity; and is strongly associated with the endorsement of misinformation and conspiracy theories. Epistemic vice explains additional variance in the endorsement of misinformation and conspiracy theories over and above demographic and related psychological concepts and shows medium to large effect sizes across outcome measures. We demonstrate that epistemic vice differs from existing psychological constructs, and show that the scale can explain individual differences in dealing with misinformation and conspiracy theories. We conclude that epistemic vice might contribute to “postfactive” ways of thinking.

**Supplementary Information:**

The online version contains supplementary material available at 10.1007/s13164-021-00562-5.

## Introduction

Epistemic vices are character traits that interfere with acquiring, maintaining, and transmitting knowledge. Social epistemologists in philosophy have hypothesized that the epistemically vicious are more susceptible to misinformation and conspiracy theories (Battaly, 2008; Cassam, 2016, 2019; Montmarquet, 1993; Roberts & Wood, 2007; Zagzebski, 1996). Moreover, whereas epistemic vice is conceptually related to psychological constructs such as personality, dogmatism, cognitive reflection and others, epistemic vice is maintained to be different from all of these constructs (Cassam, 2019). If this assumption turns out to be correct, epistemic vice may provide an important complementary explanation for individual differences in susceptibility to misinformation, conspiracist beliefs, and related pernicious beliefs .

This paper addresses the problem to make epistemic vice measurable by developing and validating the Epistemic Vice Scale (EVS), a 10-item self-assessed survey instrument. Moreover, we investigate whether epistemic vice is indeed a separate construct from various related psychological measures, and to what extent epistemic vice explains individual differences in susceptibility to misinformation and conspiracist thinking over and above demographic variables and other psychological concepts.

### Theoretical Background

Epistemic vices are character traits that interfere with acquiring, maintaining, and transmitting knowledge. Research on epistemic vices and their correlative epistemic virtues has mainly been conducted in philosophy, which has led to an emphasis on conceptual and theoretical matters (Battaly, 2008; Cassam, 2016, 2019; Montmarquet, 1993; Roberts & Wood, 2007; Zagzebski, 1996). Philosophers have provided conceptual analyses of various epistemic vices, including closed-mindedness, intellectual arrogance, and gullibility. Based on a thorough review of the literature of epistemic virtues and vices, we developed a working taxonomy of epistemic vice to aid the item generation process. Our taxonomy of epistemic vices is a consensus definition that integrates the major elements of the leading proposals that have been put forward in the literature. We decided to exclude other-regarding (Kawall, 2002) and deliberative epistemic vices (Aikin & Clanton, 2010) because they involve social rather than individual cognition, such as the sharing and joint production of knowledge. Future research could address these families of epistemic vices. Epistemic virtue and vice are thought to be associated with educational achievement (Baehr, 2013), business and financial decision-making (de Bruin, 2014), and susceptibility to conspiracy theories (Cassam, 2016).

Epistemic vices differ from cognitive defects (such as lowered IQ as a result of prenatal exposure to lead) in the sense that epistemic vices are always reprehensible, and sometimes blameworthy (Cassam, 2019). Unlike those who have a lower IQ as a result of lead poisoning, say, the bearers of epistemic vices are open to criticism for displaying epistemically vicious traits, because they are responsible either for acquiring these vices or for continuing to embody them.

Epistemic vices also differ from cognitive biases, understood in a certain way (Cassam, 2019). Consider the availability heuristic as an example of a cognitive bias. The availability heuristic is the tendency to overestimate the likelihood of events with greater “availability” in memory. More recent and more emotionally charged memories tend to be more readily available to people. The availability heuristic gets in the way of knowledge because how recent or emotionally charged a memory is does not predict the likelihood of similar events well. In contrast to epistemic vices, cognitive biases such as the availability heuristic are universal as almost everyone can be led astray by them. Cognitive biases are sometimes resistant to revision because they operate largely unconsciously.

Yet there are other cognitive biases that are either modulated by epistemic vice or can even be regarded as epistemic vices in their own right. Consider confirmation bias, the tendency to search for information that confirms your preconceptions (Klayman, 1995). Confirmation bias can be checked by conscious effort. Genuinely curious and open-minded people should therefore be less likely to undermine knowledge by falling into confirmation bias.

### Review of Relevant Scholarship

While researchers have only recently started to interrogate the empirical underpinnings of epistemic virtues and vices (Fairweather & Flanagan, 2014), psychologists have studied a number of concepts associated with epistemic vice. A number of constructs regularly investigated in psychology predict individual differences in susceptibility to fake news and conspiracy thinking. Therefore, the constructs these scales measure might overlap with epistemic vice. First, the Cognitive Reflection Test measures the tendency to override an incorrect “gut” response and engage in further reflection to find a correct answer (Sirota & Juanchich, 2018). The cognitive reflection test has also been shown to be associated with susceptibility to fake news (Pennycook & Rand, 2019, 2020) and conspiracist thinking (Swami et al., 2014). Second, scales measuring “open-minded cognition” capture the willingness to consider a variety of epistemic perspectives (Price et al., 2015), which may relate inversely to the epistemic vice of closed-mindedness, as suggested by studies of how actively open-minded thinking relates to judgements of other people’s thoughts (Baron, 2019). Third, scales measuring dogmatism capture the tendency to consider views as undeniably true, which may relate to epistemic obstinacy (Altemeyer, 2002). Dogmatism has also been shown to be predictive of belief in fake news (Bronstein et al., 2019). Fourth, scales measuring faith in intuition capture the tendency to rely on intuitive information processing, which may be epistemically inferior to more reflective styles of reasoning in some environments (Alós-Ferrer & Hügelschäfer, 2012). For instance, intuitive thinking styles have been shown to be predictive of belief in conspiracies (Swami et al., 2014).

Other constructs in psychology seem to be closely conceptually related to epistemic vice. First, scales measuring personality capture constructs such as Honesty, Conscientiousness, and Openness to Experience, which may overlap with epistemic vices (Thalmayer & Saucier, 2014). Second, scales measuring “need for closure” capture aversion to ambiguity, which may come at the cost of admitting ignorance, even to oneself (Roets & Van Hiel, 2011). Third, scales measuring “need for cognition” may relate to epistemic vice by capturing the tendency to engage in and enjoy activities that require thinking (Cacioppo et al., 1984). Fourth, scales capturing trust in experts capture the tendency to trust experts over lay people, and relate to the vice of epistemic sloppiness (Imhoff et al., 2018). Fifth, scales measuring overclaiming, the tendency to claim more knowledge than one has, may also be related to epistemic vice (Bing et al., 2011). Overclaiming scales are based on respondent’s self-ratings of their knowledge of various entities. Yet some of the proposed items do not in fact exist. Overclaiming bias, the tendency to claim knowledge about these non-existent entities, is best conceived of as a result of epistemic vice. Yet the ability to discriminate between existent and non-existent items, or accuracy, may be negatively related to the epistemic vice of sloppiness. Sixth, Rosenberg’s 10-item self-esteem scale measures feelings of self-worth, which may be thought to relate to how strongly people adhere to beliefs (Rosenberg, 1965).

Given the large number of existing psychological constructs that appear to be related to epistemic vice, one important question is whether epistemic vice is already sufficiently well measured by one or a combination of these other scales. It is possible that epistemic vice is tapping into the same psychological construct as one of the scales above and merely describes it differently, therefore scale validation should test for convergent and divergent validity with these scales.

There are some scales that try to measure epistemic vice. To date, scale construction has focussed on intellectual humility as an epistemic virtue, which is the opposite of an epistemic vice. People with intellectual humility have a sense of their fallibility, and do not unjustifiably give more weight to their own beliefs than to those of others. Intellectual humility, as Krumrei-Mancuso and Rouse define it, is “a nonthreatening awareness of one’s intellectual fallibility” (Krumrei-Mancuso & Rouse, 2016, p. 212). McElroy-Heltzel et al. (2014) put forward a 16-item scale with two factors (intellectual openness and intellectual arrogance).

Alfano et al. (2017), Leary et al. (2017) and Haggard et al., 2018 are, to our knowledge, the most recent published intellectual humility scales. Alfano et al.’s comes in self-report and informant-report versions. The authors find positive correlations between self-reports and third-party reports on their humility measure. This result provides a reason for optimism for the EVS, which relies on self-reports, in the face of potential concerns about response bias (e.g., social desirability, participant acquiescence,), as well as on the grounds that many respondents may lack the introspective insight into their cognitive lives necessary for their answers to be accurate (Bollinger & Hill, 2012). In addition to the results from Alfano and colleagues, Rowatt et al. (2006) and Landrum (2011) persuasively argue that these criticisms do not mean that self-report measures cannot provide relevant and reliable insights into epistemic virtues. While Meagher et al. (2021) find that self-assessments of intellectual humility and assessments of others are only weakly and non-significantly corelated, they argue that self-reports may in fact be more predictive of epistemic outcomes than assessments by others.

Leary et al. (2017) develops a scale of intellectual humility focused on testing whether people recognize that their beliefs might be wrong. In one study, the authors show that people high in intellectual humility were more attuned to the strength of persuasive arguments than those who were low. This gives us some reason to believe that epistemic vice may be correlated with other epistemic outcomes, like the ones we test in this study, also.

Haggard et al. (2018) develop a limitations-owning perspective, focusing on whether people properly recognize the impact of their intellectual limitations and are motivated to overcome them. The scale is based on a sophisticated philosophical analysis of intellectual humility and shows expected correlations with related constructs such as open-mindedness, dogmatism, and closed-mindedness. The authors demonstrate strong test-retest reliability over 5 months, giving some reason to believe that epistemic traits such as intellectual humility are stable character traits.

In sum, while there are many scales investigating constructs conceptually or empirically related to epistemic vice, there is not yet a scale aimed at epistemic vice directly. Developing such a measure with good psychometric characteristics, validity, and interpretability is a clear gap in the literature, as it would advance the empirical study of vice epistemology.

## Methods

In developing the Epistemic Vice Scale, we follow best practice in scale construction as a three-phase process. In the substantive validity phase reported in this section, we create an initial item pool based on a literature review of the construct to be measured and expert-review studies. In the structural validity phase, we select items based on their psychometric properties and create provisional scales (Study 1). Finally, in the external validity phase, we conduct studies to evaluate convergent, divergent, discriminant, and criterion-related validity (Study 2).

Based on a thorough review of the literature of epistemic virtues and vices, we developed a working taxonomy of epistemic vice to aid the item generation process. Our working taxonomy for item generation distinguishes five vices. Three vices pertain to three phases of information-processing: closed-mindedness pertains to the selection of evidence; sloppiness to the evaluation of evidence; and obstinacy to the formation of beliefs. Two epistemic vices concern the motivation to gain knowledge and understanding: apathy concerns the lack of a drive to gain knowledge and understanding; diffidence pertains to an overwhelming concern with avoiding embarrassment. More precisely:

- *Closed-mindedness* is the disposition to ignore certain standpoints and to be partial in appraising the reliability of sources of information.
- *Sloppiness* is the disposition to consider evidence haphazardly, shallowly, and carelessly.
- *Obstinacy* is the disposition to stick to your view even though evidence you encounter suggests otherwise.
- *Apathy* is the disposition to lack curiosity and thus fail to seek knowledge and understanding.
- *Diffidence* is the disposition to avoid social embarrassment even at the expense of shirking from the pursuit of knowledge and understanding.

This taxonomy helped us to generate items spanning a wide range of epistemic vices. However, lay no claim to having sampled the domain of epistemic vice comprehensively. As one anonymous reviewer has pointed out, we do not cover the vice of excessive malleability. The psychometric analysis below shows that not all of these conceptual distinctions correspond to (detectable) psychological differences, nor are all vices equally predictive of epistemic failings. In the structural validity phase, we show that two psychological traits underlie the epistemic vices that we investigate: indifference and rigidity. Indifference manifests itself in a lack of motivation to find the truth, and covers apathy and diffidence. Rigidity manifests itself in being insensitive to evidence once a person has made up their mind, covering closed-mindedness, sloppiness and obstinacy. This explains the simpler structure of the final Epistemic Virtue Scale, differentiating only between rigidity and indifference.

Based on our review of the literature and following guidelines for item generation, we generated 80 items potentially measuring epistemic virtue and vice. These items were drawn from examples from the literature on epistemic virtue and vice and the measurement of cognitive traits referenced above as well as directly based on the above definitions, which were extracted from the literature. We iterated the item list in a three-step process:

First, we elicited detailed feedback on the item pool through an online survey from six subject matter experts (SMEs), with expertise in epistemic virtue and vice (or “virtue epistemology” more broadly). All SMEs were professors or researchers, with Ph.D.s in philosophy or psychology, who had published and/or were working on epistemic virtue and vice. SMEs were presented with a project outline and definitions of all vices. Prospective items were grouped in sections devoted to one particular epistemic vice, and SMEs were asked to rate these items on how well they reflect a given epistemic vice and provide comments. The feedback led us to revise most items, add 15 new items, and discard 40 items. The result was a list of 55 items, with at least ten items for each vice.

Second, we asked a convenience sample of 20 people without prior knowledge of the scholarly literature on epistemic virtue and vice to rate each of the 55 items for construct fidelity and comprehension. Respondents were also given the opportunity to suggest improvements for each of the items. If respondents on average judged an item to capture another than the intended vice, we discarded or revised the item.

Third, we conducted a pilot study, experimenting with different ways of scaling the EVS. We ultimately selected 40 items as candidates for the EVS, with eight items per trait. Four items per virtue were positively keyed, and 4 negatively.

## Study 1: Structural Validity: Exploratory Factor Analysis

### Methods^1^

#### Participants and Procedure

799 participants were recruited and compensated using Amazon.com’s Mechanical Turk platform. The sample size was determined using prior power analysis, with G*power. The eligibility criteria were living in the United States and being 18 or older. 57% of participants were male. Ages ranged from the bracket 18–29 to the bracket 74 and up, with a median in the bracket from 30 to 39. The median education level achieved was a Bachelor’s degree. 68% of respondents had a Bachelors or higher level of education. The median annual household income fell in the bracket of $50,000 to $74,999. These descriptive statistics appear to be roughly in line with the population of the United States, with men and highly-educated people somewhat over-represented in our sample (in 2019, 32% had a Bachelor’s degree or higher level of education, according to the estimates based on the 2019 vintage of the US census).

#### Measures

The 40 candidate items of the EVS introduced above were administered in random order using a five-point Likert scale (anchored on “strongly disagree” through “strongly agree”). Table 1 contains the full item list. We collected demographic information as described above. In order to gauge the associations of items with outcomes, we collected data on the propensity of respondents to endorse conspiracy theories, using an established measure from political science (Oliver & Wood, 2014). Participants were presented with five conspiracy theories in random order and asked to respond to all items on a five-point scale (“Definity false”, “Probably false”, “Don’t know”, “Probably true”, “Definitely true”). Table 2 presents descriptive statistics. 40% of respondents thought at least one conspiracy theory was probably true or definitely true (α = .91). The aggregate measure takes the mean of the five items. “Don’t know” responses were excluded from the analysis, because they could either be read as evidence of epistemic vice since participants fail to reject the theories, or as epistemic virtue because suspending judgment can be a sign of humility. The results are qualitatively the same if don’t know responses are included in the analysis.

**Table 1:** Item pool

Name	Item	Key
Apathy1	I want to understand things.	negative
Apathy2	I want to know the reasons why.	negative
Apathy3	I am curious to learn new things.	negative
Apathy4	I enjoy gaining knowledge.	negative
Apathy5*	**I am not very interested in understanding things.**	positive
Apathy6*	**I am not so interested in the reasons why.**	positive
Apathy7*	**I am not particularly curious to learn new things.**	positive
Apathy8*	**I do not much enjoy gaining knowledge.**	positive
Closed1	I gather many different perspectives before I make up my mind.	negative
Closed2	I consider the views of people I disagree with.	negative
Closed3	Being open-minded is a valuable trait.	negative
Closed4	I am open-minded towards viewpoints different from my own.	negative
Closed5*	I mostly consider a topic from my preferred perspective to make up my mind.	positive
Closed6	I pay less attention to the views of people I disagree with.	positive
Closed7*	**It’s more important to have a stable worldview than to be open-minded.**	positive
Closed8*	I am not very open-minded towards viewpoints different from my own.	positive
Sloppiness1	I carefully think things through.	negative
Sloppiness2	I think through the relevant factors before making up my mind.	negative
Sloppiness3	I weigh the pros and the cons when I make up my mind.	negative
Sloppiness4	I reason carefully and critically before making decisions.	negative
Sloppiness5	I tend not to think things through at great length.	positive
Sloppiness6*	**I make up my mind without much fuss about the many factors that may affect an issue.**	positive
Sloppiness7	I do not dwell much on the pros and the cons when I make up my mind.	positive
Sloppiness8*	**I tend to make decisions based on my gut feeling.**	positive
Obstinacy1	I could be wrong about many things.	negative
Obstinacy2	I have a realistic sense of what I know.	negative
Obstinacy3	The more I know about an issue, the more confident I become of my opinions.	negative
Obstinacy4	How sure I am about my view depends on the strength of my evidence.	negative
Obstinacy5*	I think I am right about most things.	positive
Obstinacy6*	**I tend to be too confident in my opinions.**	positive
Obstinacy7*	**I often have strong opinions about issues I don’t know much about.**	positive
Obstinacy8*	**I tend to feel sure about my views even if I don’t have much evidence.**	positive
Diffidence1	I follow an argument where it leads, even if the conclusion is unpopular.	negative
Diffidence2	To get to the bottom of an issue, I even ask questions that could make me look stupid.	negative
Diffidence3	If I do not understand an answer, I keep asking until I understand.	negative
Diffidence4	I ask questions even if they reveal my ignorance.	negative
Diffidence5	I am afraid to adopt an unpopular opinion.	positive
Diffidence6*	I avoid asking questions that could make me look stupid.	positive
Diffidence7*	If I do not understand an answer, I sometimes pretend I do.	positive
Diffidence8*	I avoid asking questions that might reveal my ignorance.	positive

Key: positive: “agree” indicates vice; negative: “disagree” indicates vice.

*= included in the confirmatory analysis. **Bold** = included in the final scale

**Table 2:** Descriptive Statistics for the measure of Conspiracist Thinking

#	Item	Mean	SD	Skew	Kurtosis
1	The US invasion of Iraq was not part of a campaign to fight terrorism but was driven by Jews in the U.S. and Israel.	1.97	1.27	1.11	−0.19
2	Certain U.S. government officials planned the attacks of September 11, 2001, because they wanted the United States to go to war in the Middle East.	2.05	1.35	0.96	−0.62
3	President Barack Obama was not really born in the United States and does not have an authentic Hawaiian birth certificate.	1.92	1.37	1.17	−0.23
4	The financial crisis of 2008/09 was secretly orchestrated by a small group of Wall Street bankers to extend the power of the Federal Reserve and further their control of the world’s economy.	2.21	1.37	0.76	−0.95
5	Billionaire George Soros is behind a hidden plot to destabilize the American government, take control of the media, and put the world under his control.	2.05	1.37	1.00	−0.53

SE = 0.05

### Results

We conducted an initial exploratory factor analysis based on all 40 items. The analysis revealed that whether items were keyed positively or negatively completely determined the factor structure. When extracting two factors, all negatively keyed items had their primary loadings on one factor, and all positively keyed items on the other, with the exception of one item which did not have large loadings on either factor.

There is a tradeoff between keeping items that are keyed in different directions or including only items keyed in one direction (Weijters et al., 2013). Using items keyed in both directions introduces acquiescence, careless responding, and confirmation bias. To inform our choice, we considered correlations between conspiracist thinking and positive and negatively keyed items, respectively. We chose this test because there is a strong theory-based reason to expect that epistemic vice is correlated with conspiracist thinking (Cassam, 2016). If there is a strong difference in the extent to which positively and negatively keyed items correlate with conspiracist thinking, this is a strong reason to suspect that response biases are determining the factor structure. Indeed, the Pearson correlation of the mean of positively keyed items with the conspiracy score was .64 (*p* < 0.01), whereas the correlation of the mean of the negatively keyed items with the conspiracy score was almost three times lower (r = .22, *p* < 0.01). Table S1 shows correlations for each of the 40 items and the conspiracy score. The table shows that positively keyed items (items 5–8 in each category) correlate more strongly than negatively keyed items (items 1–4 in each category). Based on this result, we decided to conduct further analysis using the positively keyed items only.

Parallel analysis of positively keyed items suggested extraction of up to four factors. Table S2 in the online supplemental material shows the factor loadings and fit statistics for a four factor analysis, using oblimin rotation. The factor analysis shows excellent fit (RMSR = 0.02, RMSEA = 0.03, TLI = 0.98). However, a look at the loadings suggests several ways of improving the scale. Closed6 (“I pay less attention to the views of people I disagree with”) is the only item loading on the fourth factor. Closed6 is also the positive item with the lowest correlation of all positively keyed items with the conspiracy score. Finally, the SS loading of Factor 4 is low (1.15). We decided on that basis to remove Closed6 from the scale. We also removed Sloppiness5, Sloppiness7, and Diffidence5 because each item has large cross-loadings. Sloppiness5 and Diffidence5 are also problematic because they load below .4 on their primary factor.

We conducted a parallel analysis for the new item set. Factor analysis using oblimin rotation shows that the four-factor model has moderate fit, RMSR = 0.04, RMSEA = 0.08, TLI = 0.89. The analysis suggested the extraction of up to three factors, and the scree plot suggested two. The four-factor solution had worse fit than both the three-factor and two-factor solutions.

In order to ensure that the final scale was short enough for inclusion in studies using multiple instruments, we used methods from item response theory to further shorten the scale. Figs. S1 and S2 in the online supplemental material show the item characteristic curves for the three factors extracted based on the revised item set. We removed items that provide little information across the ability spectrum. For the first factor, Apathy8, Closed8, and the Diffidence items stand out as providing little information. For the second factor, we decided to remove Obstinacy5 and Closed5 because they provide little information.

Parallel analysis for the newly-revised item set suggested a two-factor solution. Table 3 shows the factor loadings and fit statistics for a two-factor analysis, using oblimin rotation. This model has excellent fit: RMSR = 0.02, RMSEA = 0.03, TLI = 0.99. There are no cross loadings > .2, and all factor loadings are above .5, indicating a reasonably clean factor structure.

**Table 3:** Factor loadings of two factor solution based on final item list

	Factor1	Factor2
Apathy5	0.00	0.76
Apathy6	0.08	0.73
Apathy7	0.06	0.75
Apathy8	−0.06	0.90
Closed7	0.62	0.10
Sloppiness6	0.58	0.10
Sloppiness8	0.75	−0.07
Obstinacy6	0.61	0.01
Obstinacy7	0.55	0.15
Obstinacy8	0.78	−0.04
SS loadings	2.29	2.52
Proportion Var	0.26	0.25
Cumulative Var	0.28	0.52

“SS loadings” = Sum of squared loadings; “Proportion Var” = proportion of variance explained by each factor, “Cumulative Var” = “cumulative proportion of variance explained.

The resulting scale consists of 10 items (α = .90): with two subscales: indifference to truth (items loading on factor 1) consists of 4 items (α = .88), and rigidity(items loading on factor 2) consists of 6 items (α = .84).

### Discussion

The extraction of two factors based on the revised item set produces a clean and interpretable factor structure. Factor 1 covers apathy and diffidence. We call the subscale based on this factor *indifference to truth*. Indifference manifests itself in a lack of motivation to find the truth, and covers apathy and diffidence. Factor 2 has items related to closed-mindedness, sloppiness, and obstinacy loading on it. Examination of the items reveals that they are all indicative of a certain *rigidity in thinking*. Thus, rigidity manifests itself in being insensitive to new evidence once one’s mind is made up. Hence factor analysis suggests that the conceptual differentiation by phase of information processing does not correspond to a measurable psychological difference, but rather reveals rigidity as a common underlying factor.

## Study 2: Confirmatory Factor Analysis, Convergent/Divergent Analysis, and Unique Variance

### Methods^2^

#### Participants and Procedure

998 participants were recruited and compensated using Amazon.com’s Mechanical Turk platform. Sample size was determined by prior power analysis using G*Power. The eligibility criteria were living in the United States and being 18 or older. Ages ranged from the bracket 18–29 to the bracket 74 and up, with a median in the 29 to 39 bracket. 63% of participants were male. Median education completed was a Bachelor’s Degree; 68% had a Bachelors or higher level of education. The median annual household income fell in the bracket of $50,000 to $74,999. 55% of respondents were married; 34% had never married; 7% were divorced; 2% separated; and 2% widowed. 38% of respondents identified as Republican to various degrees; 47% as Democrats; and 15% as Independent. 74% of respondents were White / Caucasian; 12% were Black or African American; 5% Hispanic; 7% Asian or Pacific Islander; and 2% American Indian or Alascan Native. 49% of respondents rated religion as not at all important or not very important; 18% as moderately important, and 33% as important or extremely important. Hence our sample is more male, more White / Caucasian, and more educated than the US as a whole, and probably also slightly less religious and less Republican, although different ways of eliciting this information make comparisons difficult.

#### Measures

The 16 items of the EVS that we identified as promising based on the exploratory factor analysis above were administered in random order using a five-point Likert scale (anchored on “strongly disagree” through “strongly agree”) -- the ten items used in the final exploratory factor analysis, as well as the six items removed last, to see whether they perform better in this study. The items are marked with an asterix in Table 1 above.

Each participant answered demographic questions about age, gender, race, education, household income, and marital status.

To evaluate unique variance, we administered three instruments. First, administered the conspiracy instrument we used in study 1 (α = 0.91, items = 5) Descriptive statistics are in Table 4.

**Table 4:** Descriptive Statistics for the measure of Conspiracist Thinking

#	Item	Mean	SD	Skew	Kurtosis
1	The US invasion of Iraq was not part of a campaign to fight terrorism but was driven by Jews in the U.S. and Israel.	2.25	1.29	0.61	−0.91
2	Certain U.S. government officials planned the attacks of September 11, 2001, because they wanted the United States to go to war in the Middle East.	2.30	1.33	0.54	−1.09
3	President Barack Obama was not really born in the United States and does not have an authentic Hawaiian birth certificate.	2.14	1.36	0.76	−0.89
4	The financial crisis of 2008/09 was secretly orchestrated by a small group of Wall Street bankers to extend the power of the Federal Reserve and further their control of the world’s economy.	2.42	1.31	0.34	−1.27
5	Billionaire George Soros is behind a hidden plot to destabilize the American government, take control of the media, and put the world under his control.	2.36	1.35	0.49	−1.10
6*	California’s economy is the biggest of all US states	3.83	1.06	−0.80	0.07
7*	The U.S. gained independence in 1912.	1.70	1.23	1.46	0.61

SE = 0.04

*= control questions added to the confirmatory study, not included in the construction of the mean score

Second, to demonstrate real-world relevance of the EVS, we administered a 12-item measure of Covid-19-related misinformation based on the “myth-busting” page of the World Health Organisation^3^ (α = 0.94). Items concern essential information about Covid-19 for the public, e.g. “Being able to hold your breath for 10 seconds or more without coughing or feeling discomfort means you are free from the coronavirus disease”, “Spraying and introducing disinfectant into your body will protect you against COVID-19”, and “Regularly rinsing your nose with saline helps prevent infection with the new coronavirus” (see Table 5 for all items and descriptive statistics). Participants were asked to respond to all items in random order on a five-point scale (“Definity false”, “Probably false”, “Don’t know”, “Probably true”, “Definitely true”). The aggregate measure takes the mean of the responses to the first three items. “Don’t know” responses were excluded from the analysis, for the same reason as in study 1 that the response might either indicate epistemic virtue or vice.

**Table 5:** Descriptive statistics for Covid-19 misinformation items

#	Item	Mean	SD	Skew	Kurtosis
1	Adding pepper to your meals prevents COVID-19.	1.80	1.25	1.36	0.44
2	COVID-19 can be transmitted through houseflies.	2.08	1.19	0.84	−0.37
3	Spraying and introducing disinfectant into your body will protect you against COVID-19.	1.85	1.30	1.19	−0.13
4	Drinking methanol, ethanol or bleach prevents COVID-19.	1.62	1.17	1.73	1.60
5	5G mobile networks spread COVID-19.	1.70	1.12	1.50	1.11
6	Exposing yourself to the sun or to temperatures higher than 77 °F prevents the Coronavirus disease.	2.12	1.30	0.78	−0.77
7	Catching Covid-19 means you will have it for life.	2.03	1.21	0.90	−0.35
8	Being able to hold your breath for 10 s or more without coughing or feeling discomfort means you are free from the Coronavirus disease.	2.08	1.35	0.87	−0.73
9	Hand dryers are effective in killing Coronavirus.	2.09	1.27	0.87	−0.49
10	Regularly rinsing your nose with saline helps prevent infection with Covid-19.	2.27	1.30	0.60	−0.92
11*	Some people infected with Coronavirus experience no symptoms.	4.50	0.90	−2.25	5.10
12*	Older people are more likely to die due to an infection with Covid-19.	4.43	0.86	−1.80	3.24

*N* = 998. Scale = 1–5. SE = 0.04 for items 1–10; SE = 0.03 for items 11 and 12 * = control items not included in calculation of Covid-19 misinformation score.

Third, we administered a measure of fake-news endorsement related to the Covid-19 pandemic (α = 0.91, items = 4). Participants were presented with screenshots of five online news articles related to Covid-19 in random order, four fake and one legitimate (see Table 6 and Figs. S3 to S7 in the online supplemental material for all items and descriptive statistics). Participants were asked to respond to the statement “The article displayed above is credible” on a five-point agree/disagree scale. The four fake news items were picked from prominent fake news sources. They spread claims about evidence that the new Coronavirus is a bioweapon; that the pandemic is deadlier due to the introduction of 5G technology; and that reporting on Covid-19 is a ploy to distract from the shady dealings of certain politicians. From a content perspective, they are quite similar to the Covid-19 misinformation instrument. But this instrument differs by eliciting how much credence respondents give to fake news *articles* advancing such claims, rather than the *claims* themselves.

**Table 6:** Fake news items

#	Item	Mean	SD	Skew	Kurtosis
1	The Hidden Back Story Emerges	2.46	1.39	0.39	−1.29
2	Is Coronavirus a Manufactured Bioweapon?	2.24	1.36	0.68	−0.95
3	Full Transcript of Smoking Gun Bombshell Interview	2.47	1.42	0.39	−1.32
4	Did 5G Make Coronavirus Deadlier?	2.19	1.36	0.69	−1.01
5*	Lost Sense of Smell Clue to Coronavirus Infection	4.04	0.99	−1.21	1.29

N = 998. Scale = 1–5. SE = 0.04 for items 1–4; SE = 0.03 for item 5

*= control item not included in calculation of fake news score

To evaluate convergence and divergence with related constructs, we administered 10 scales: First, we measured all dimensions of the Big Six personality model using the 24-item QB6, α(Honesty) = .56, α(Agreeableness) = .62, α(Emotionality) = .62, α(Extroversion) = .60, α(Conscientiousness) = .68, α(Intellect) = .43 (Thalmayer & Saucier, 2014). Second, a seven-item version of the Cognitive Reflection Test, α = .80 (Sirota & Juanchich, 2018). Third, Rosenberg’s 10-item self-esteem scale, α = .85 (Rosenberg, 1965). Fourth, a 15-item scale to measure need for closure, α = .89 (Roets & Van Hiel, 2011). Fifth, a 18-item scale to measure need for cognition, α = .93 (Cacioppo et al., 1984). Sixth, a 15-item scale to measure faith in intuition, α = .93 (Alós-Ferrer & Hügelschäfer, 2012). Seventh, the general version of a 6-item scale on open-minded cognition, α = .77 (Price et al., 2015). Eighth, a 20-item dogmatism scale, α = .90 (Altemeyer, 2002). Ninth, a 6-item scale measuring trust in experts, adapted from Imhoff et al. (2018), α = .74 (see Table S3 for items). Tenth, a 25-item scale to measure overclaiming, α = .87 (Bing et al., 2011).

We measure religiosity by asking respondents how important religion is to them, on a five-point scale from “not at all important” to “extremely important.”

We measure political partisanship by asking participants whether they “consider themselves a Republican, a Democrat, an Independent, or what?” Responses are “Strong Democrat”, “Moderate Democrat”, “Lean Democrat”, “Lean Republican”, “Moderate Republican”, “Strong Republican”, “Independent”, “Other”, and “Prefer not to say”.

Before conducting the study, we recorded our hypotheses in the process of pre-registration.^4^ We expected the EVS to be strongly positively correlated with all three outcome measures. We expected that the scales we included for convergent/divergent validity would show the following correlations: positive correlations for the scales measuring faith in intuition, dogmatism, overclaiming (accuracy), and need for closure; negative correlations for all other scales: personality, cognitive reflection, self-esteem, trust in experts, need for cognition, open-minded cognition, and the accuracy measure on the overclaiming scale. We expected religiosity to be positively related to the EVS, and education to be related negatively. We expected that all correlations will be moderate, which would establish epistemic vice as a distinct construct. Our most important hypothesis was this: *EVS can explain additional variance with regard to all three outcome metrics, over and above the demographic information, the scales included for divergent validity, and political affiliation and religiosity.*

### Results

#### Confirmatory Factor Analysis

In the CFA we included the ten items that were used in the final exploratory factor analysis in Study 1. We also experimented with the inclusion of some or all of the six items cut last in Study 1, but decided to discard them because the models had less good fit. The models were examined according to several indices: the robust versions of the comparative fit index (CFI), the root mean square error of approximation (RMSEA), and the standardized root mean square residual (SRMR). CFI ranges from 0 to 1, with values closer to 1 indicating better fit, and reflects the proportion of improvement in fit relative to the null (independence) model. RMSEA and SRMR are measures of absolute fit, that is, how well on average the correlation matrix has been reproduced by the model. According to Hu and Bentler (Hu & Bentler, 2009), CFI should not be smaller than .95, RMSEA should not be more than .06, and SRMR should not be more than .08. The two-factor solution met Hu and Bentler’s standards, χ^2^(34) = 150, CFI = .0.98, RMSEA = .06, SRMR = .03 – though note that the RMSEA value is at the cutoff point. See Table 7 for the final 2-factor solution with ten items and standardized estimates.

**Table 7:** The final version of the epistemic vice scale with CFA estimates

Item	Description	Indifference	Rigidity
Apathy5	I am not very interested in understanding things.	0.82	
Apathy6	I am not so interested in the reasons why.	0.82	
Apathy7	I am not particularly curious to learn new things.	0.84	
Apathy8	I do not much enjoy gaining knowledge.	0.85	
Closed7	It’s more important to have a stable worldview than to be open-minded.		0.68
Sloppiness6	I make up my mind without much fuss about the many factors that may affect an issue.		0.68
Sloppiness8	I tend to make decisions based on my gut feeling.		0.62
Obstinacy6	I tend to be too confident in my opinions.		0.63
Obstinacy7	I often have strong opinions about issues I don’t know much about.		0.71
Obstinacy8	I tend to feel sure about my views even if I don’t have much evidence.		0.71

Factor loading estimates are STYDX. N = 998

The scale has very similar reliability to the reliability reported in study 1. Reliability of the Epistemic Vice Scale is high (α = .90, items = 10), as are the reliability of the indifference to truth subscale (α = .90, items = 4), and rigidity subscale (α = .83, items = 6).

#### IRT Analysis

We supplemented the confirmatory factor analysis with analyses from the perspective of item response theory (IRT). IRT is used for investigating item and test properties; it assumes a latent trait or ability that is a function of both the participants’ responses and the properties of the items. Thus, IRT allows us to estimate both an individual’s trait level and the relevant item parameters. The goal was to estimate the overall reliability of the measure in a way that is distinct from the classical testing theory approach. We used a graded response model implemented in the ltm package in the R statistical language (Rizopoulos, 2006).

**Fig. 1:**
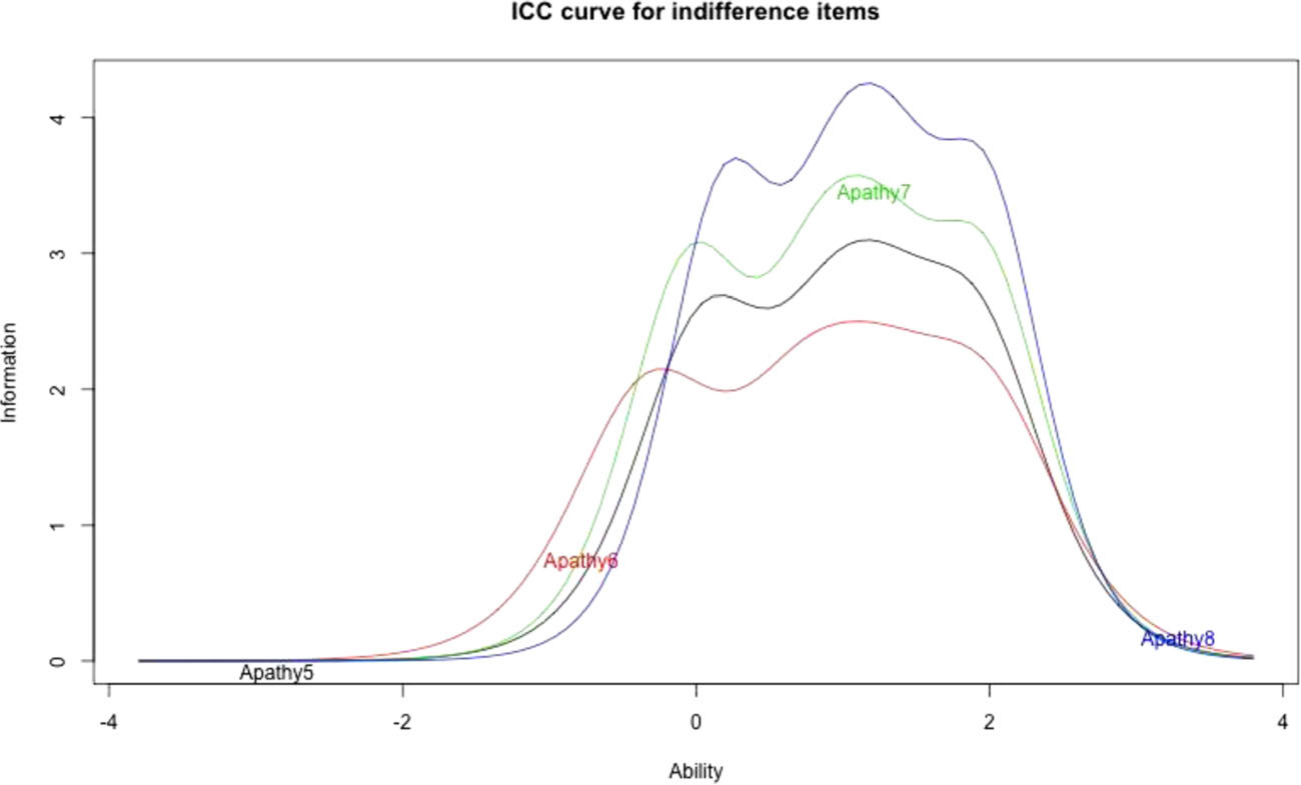
Item characteristic curves for indifference items

**Fig. 2:**
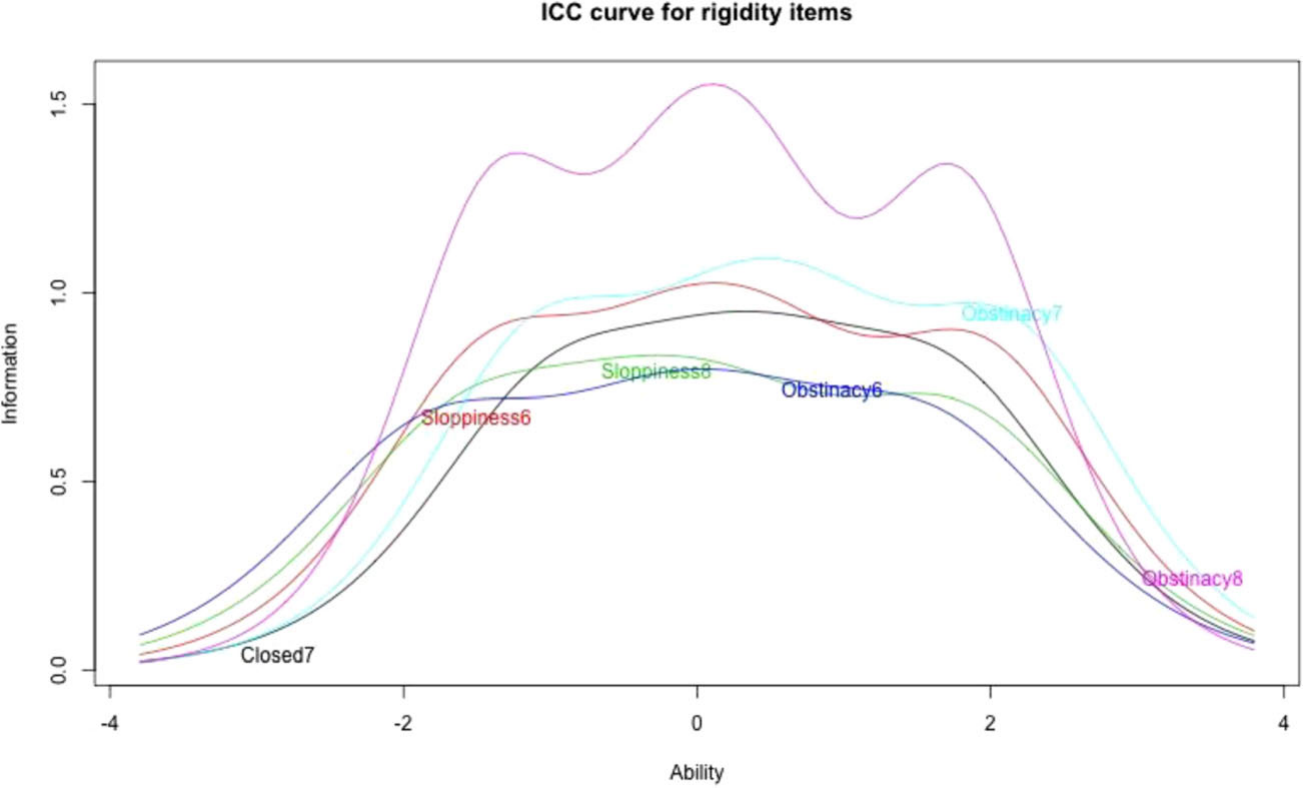
Item characteristic curves for rigidity items

Figures 1 and 2 show the item characteristic curves for the indifference and rigidity subscales, respectively. Table 8 gives threshold parameters and item slopes for items. Item slopes (column a) are discrimination parameters, which are particularly interesting because they describe an item’s ability to differentiate between participants having levels of the latent trait above or below the item’s location. Threshold parameters (b) can be considered cut-off points on the latent trait’s continuum. Respondents with the given level of the latent trait are equally likely to select the respective response category rather than the next higher response category. Consider the indifference items. One thing to note is that threshold parameters are skewed towards the positive end of the latent trait. This means that items provide more information on people who are more indifferent. While it would be desirable for items to differentiate well across the whole spectrum of the latent trait, for the purposes of identifying epistemically vicious individuals, differentiation toward the positive end of the spectrum is most important. The slope parameters are reasonably similar across items, supporting scoring of the scale as an unweighted sum. Considering the rigidity items, we find that threshold parameters are more balanced across the latent trait spectrum, compared to the indifference items, and that like in the case of the indifference items, item slopes are fairly similar.

**Table 8:** Item parameter estimates

Indifference					
	b_1_	b_2_	b_3_	b_4_	a
Apathy5	0.06	0.94	1.26	1.90	3.18
Apathy6	−0.31	0.75	1.25	1.94	2.86
Apathy7	−0.04	0.86	1.25	1.96	3.43
Apathy8	0.20	0.97	1.32	1.97	3.74
Rigidity					
	b_1_	b_2_	b_3_	b_4_	a
Closed7	−0.97	0.06	0.64	1.71	1.75
Sloppiness6	−1.40	−0.14	0.47	1.93	1.83
Sloppiness8	−1.56	−0.46	0.22	1.77	1.64
Obstinacy6	−1.77	−0.31	0.29	1.59	1.61
Obstinacy7	−1.06	0.19	0.78	2.11	1.89
Obstinacy8	−1.36	−0.11	0.36	1.78	2.27

*b indicates a threshold parameter, a indicates slope*

**Fig. 3:**
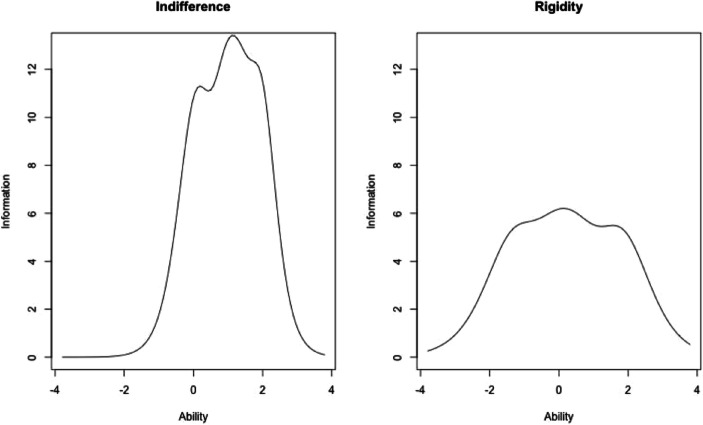
Test information function for indifference and rigidity items

Figure 3 shows the test information functions for the indifference and rigidity subscales. Consistent with the analysis above, the indifference subscale is particularly informative towards the positive end of the spectrum. The items measuring rigidity are more evenly distributed over the center of the latent trait.

#### Convergent/Discriminant Validity

We analyzed the extent to which the scale displayed convergent and discriminant validity. Table 9 shows correlation coefficients between all measures. Table S4 provides a detailed correlation table for key covariates. The first four columns show correlations with the four outcome metrics we are testing: Covid-19 misinformation, conspiracist thinking, susceptibility to fake news, and overclaiming bias. People who are susceptible to conspiracist thinking are also likely to believe Covid-19 misinformation, endorse fake news, and overclaim, as indicated by the high correlations between the four instruments.

**Table 9:**
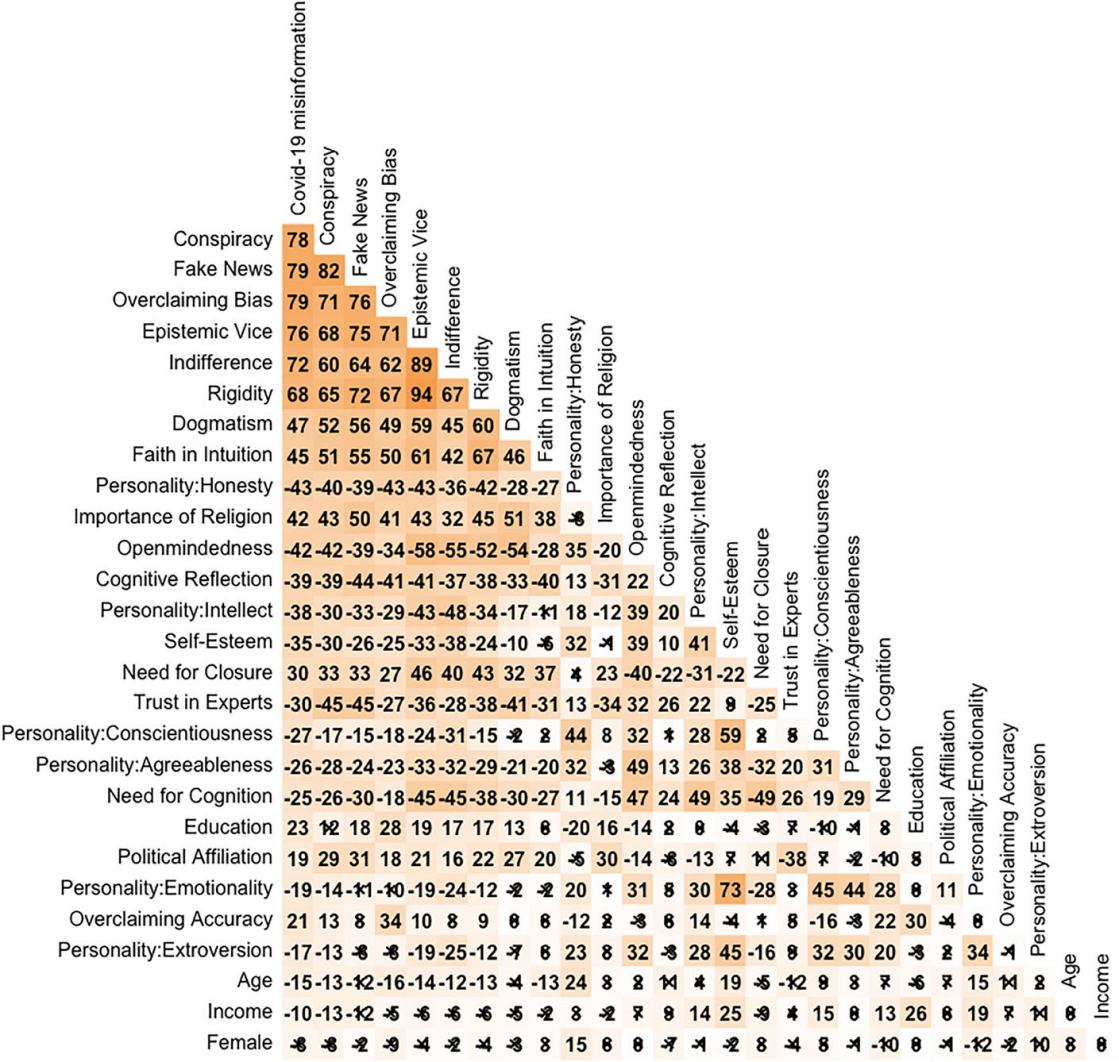
Means, standard deviations, and correlations with confidence intervals.

The table shows correlations between covariates in percentages (pairwise Pearson correlations). The shade indicates strength of correlation (absolute value). Correlations are significant at *p* < 0.01, unless crossed out (2-tailed).

Epistemic vice is strongly associated with all four instruments. Measures for competing explanations are less strongly associated with any of the outcome metrics. The measure with the next-highest correlation, dogmatism, shows substantially lower correlations.

We conceptually replicate the findings of Stanley et al. (2020) and Pennycook et al. (2020) that the Cognitive Reflection Test predicts acceptance of Covid-19 misinformation (their outcome variable was measured slightly differently, but the headline result is the same). Yet the absolute value of the correlation coefficient of cognitive reflection with Covid-19 misinformation is only about half of the correlation coefficient of epistemic vice with Covid-19 misinformation. This gives epistemic vice a fairly strong lead over alternative measures.

The associations between epistemic vice and other measures all have the expected sign, with the exception of education. We expected that higher levels of formal education would be associated with lower readiness to endorse fake news. Yet the opposite is the case. The finding that more formal education is not an important determinant of epistemic virtue and vice is however consistent with what Haggard et al. (2018) find in their validation study.

No correlation of epistemic vice or its subscales with other scales is so high as to suggest that the EVS simply replicates an existing scale. However, the EVS may still be tapping into the same latent construct as some of the other scales that it is correlated with. If so, however, there is evidence that the EVS measures that common latent trait better, because it is correlated more strongly with outcome measures. Consider for instance faith in intuition, the scale that the EVS and its subscales is most strongly correlated with (r ranges between .45 and .60). Faith in intuition is correlated to a much smaller extent with the outcome metrics (r ranges between .47 and .56) than epistemic vice (r ranges between .68 and .76).

#### Unique Variance

The correlations between epistemic vice and some established psychological scales such as dogmatism, faith in intuition, and the cognitive reflection test raise a question about whether the epistemic vice predicts outcome variables above and beyond what these scales can predict. Epistemic vice could just be a combination of existing constructs, making a new scale less useful.

Given the large number of existing psychological constructs that appear to be related to epistemic vice, one important question is whether epistemic vice is already sufficiently well measured by one or a combination of these other scales. It is possible that epistemic vice is tapping into the same psychological construct as one of the scales above and merely describes it differently. We test this by conducting hierarchical regressions to investigate how much variance in outcomes such as the propensity to endorse misinformation or conspiracy theories the EVS can explain in addition to these existing measures.

To test this, we performed hierarchical regressions where we tested how much variance in outcome variables epistemic vice predicts above and beyond what other scales and demographic variables predict.

Table 10 shows the summary results of the second and final step of a series of hierarchical regressions. The dependent variables were Covid-19 misinformation, conspiracist thinking, fake news, and overclaiming bias, respectively. Each row of the table represents the summary results of Step 2 of a separate hierarchical regression. For Step 1, the variables listed in the column “Model” were entered as a block. For Step 2, epistemic indifference and epistemic rigidity were entered as a block. All continuous predictors as well as the dependent variables are mean-centered and scaled by one standard deviation. However, adding the epistemic vice scales increases R^2^ substantially across all models and all dependent variables. Consequently, epistemic vice explains substantial variance over and above each scale for each outcome variable. Moreover, the effect size as measured by the regression coefficients of epistemic indifference and epistemic rigidity is large across models and outcome variables (epistemic indifference: min = .29, max = 53, mean = .37; epistemic rigidity: min = .28, max = .68, mean = .48).

**Table 10:** Hierarchical regressions

**Covid-19 Misinformation**							
Model	R^2^ (Step 1)	ΔR^2^	ΔF	ß Indiff.	t-test Indiff.	ß Rigidity	t-test Rigidity
Education, Age, Female, Income, Marital Status, Ethnicity	0.29	0.34	434.42***	0.45	16.38***	0.28	9.83***
Dogmatism	0.22	0.37	442.01***	0.48	17.18***	0.32	10.31***
Faith in intuition	0.21	0.38	457.20***	0.48	17.40***	0.33	9.85***
All six personality traits	0.29	0.32	397.65***	0.44	14.78***	0.32	10.99***
Importance of religion	0.17	0.42	513.47***	0.47	17.28***	0.30	10.39***
Open-mindedness	0.18	0.41	489.40***	0.49	16.90***	0.37	12.87***
Cognitive reflection	0.15	0.44	540.99***	0.46	16.55***	0.33	11.98***
Self-esteem	0.12	0.47	579.45***	0.44	15.41***	0.36	13.09***
Need for closure	0.09	0.50	598.58***	0.49	17.56***	0.37	13.13***
Trust in experts	0.09	0.50	598.76***	0.48	17.30***	0.34	11.98***
Need for cognition	0.06	0.54	669.68***	0.53	18.60***	0.37	13.69***
Political affiliation	0.09	0.50	609.77***	0.47	16.98***	0.34	12.16***
Overclaiming accuracy	0.04	0.56	705.89***	0.47	17.56***	0.35	12.83***
All measures combined	0.60	0.09	130.87***	0.38	12.88***	0.18	5.15***
**Conspiracy**							
Model	R^2^ (Step 1)	ΔR^2^ (Step 2)	ΔF	ß Indiff.	t-test Indiff.	ß Rigidity	t-test Rigidity
Education, Age, Female, Income, Marital Status, Ethnicity	0.26	0.25	249.81***	0.33	8.54***	0.46	11.44***
Dogmatism	0.27	0.21	206.97***	0.34	8.98***	0.43	9.98***
Faith in intuition	0.26	0.22	207.60***	0.37	9.69***	0.42	8.87***
All six personality traits	0.23	0.25	235.87***	0.34	7.94***	0.49	11.84***
Importance of religion	0.18	0.30	287.80***	0.35	9.21***	0.47	11.64***
Open-mindedness	0.17	0.29	270.85***	0.34	8.36***	0.55	13.64***
Cognitive reflection	0.15	0.33	312.73***	0.33	8.40***	0.52	13.26***
Self-esteem	0.09	0.38	359.53***	0.31	7.75***	0.56	14.51***
Need for closure	0.11	0.36	327.59***	0.36	8.98***	0.55	13.71***
Trust in experts	0.20	0.31	311.73***	0.35	9.31***	0.46	11.89***
Need for cognition	0.07	0.40	371.22***	0.39	9.55***	0.57	14.53***
Political affiliation	0.11	0.38	360.09***	0.35	9.05***	0.52	13.36***
Overclaiming accuracy	0.02	0.45	420.75***	0.36	9.22***	0.55	14.22***
All measures combined	0.59	0.03	33.72***	0.25	6.27***	0.14	3.01***
**Fake News**							
Model	R^2^ (Step 1)	ΔR^2^ (Step 2)	ΔF	ß Indiff.	t-test Indiff.	ß Rigidity	t-test Rigidity
Education, Age, Female, Income, Marital Status, Ethnicity	0.28	0.32	384.25***	0.30	8.70***	0.57	15.91***
Dogmatism	0.31	0.27	321.87***	0.32	9.29***	0.54	14.26***
Faith in intuition	0.31	0.27	313.91***	0.34	10.04***	0.53	12.74***
All six personality traits	0.24	0.33	388.82***	0.34	8.97***	0.60	16.33***
Importance of religion	0.25	0.35	426.70***	0.32	9.71***	0.55	15.53***
Open-mindedness	0.16	0.41	468.65***	0.36	9.77***	0.68	19.03***
Cognitive reflection	0.20	0.39	468.34***	0.29	8.46***	0.61	17.82***
Self-esteem	0.07	0.50	567.96***	0.32	8.79***	0.66	19.07***
Need for closure	0.11	0.45	513.96***	0.34	9.55***	0.66	18.61***
Trust in experts	0.20	0.40	488.08***	0.32	9.62***	0.58	16.72***
Need for cognition	0.09	0.47	539.57***	0.35	9.61***	0.67	19.09***
Political affiliation	0.13	0.46	545.98***	0.32	9.41***	0.62	17.98***
Overclaiming accuracy	0.01	0.56	633.87***	0.33	9.59***	0.66	18.99***
All measures combined	0.64	0.05	78.39***	0.27	7.39***	0.31	7.22***
**Overclaiming Bias**							
Model	R^2^	ΔR^2^	ΔF	ß Indiff.	t-test Indiff.	ß Rigidity	t-test Rigidity
Education, Age, Female, Income, Marital Status, Ethnicity	0.33	0.25	279.31***	0.29	9.13***	0.40	12.03***
Dogmatism	0.24	0.28	279.87***	0.33	9.92***	0.45	12.14***
Faith in intuition	0.25	0.26	264.53***	0.34	10.45***	0.42	10.50***
All six personality traits	0.25	0.28	298.38***	0.34	9.67***	0.44	12.68***
Importance of religion	0.17	0.35	352.25***	0.33	10.01***	0.45	13.01***
Open-mindedness	0.11	0.40	407.56***	0.38	11.01***	0.54	16.11***
Cognitive reflection	0.17	0.35	367.88***	0.30	9.17***	0.47	14.33***
Self-esteem	0.06	0.44	445.19***	0.33	9.38***	0.51	15.48***
Need for closure	0.07	0.44	440.57***	0.35	10.52***	0.53	15.74***
Trust in experts	0.07	0.43	435.53***	0.34	10.14***	0.51	14.85***
Need for cognition	0.03	0.50	522.06***	0.40	11.98***	0.54	16.53***
Political affiliation	0.09	0.43	430.77***	0.32	9.75***	0.50	14.72***
Overclaiming accuracy	0.11	0.46	546.23***	0.33	10.67***	0.49	16.03***
All measures combined	0.65	0.05	70.19***	0.26	8.03***	0.21	5.69***

This table shows summary results of the final second step of a series of hierarchical regressions. The dependent variables are susceptibility to Covid-19 misinformation, endorsement of conspiracy theories, susceptibility to fake news, and overclaiming bias, respectively. For the first step, the variables mentioned in the Model column were entered as a block. The R^2^ column shows variance explained in the first step of the regression (without epistemic vice). Each subsequent column provides results of the second step of a different hierarchical regression. For the second step, epistemic indifference and epistemic rigidity were added to the respective model as a block. *ΔR*^*2*^ shows the difference in R^2^ between the first and the second stage. *ΔF* shows the F-statistic for the difference between the two models. *ß Indiff.* and *ß Rigidity* show the coefficient of epistemic indifference and epistemic rigidity in the final step of the regression, respectively. All continuous predictors as well as the dependent variables are mean-centered and scaled by 1 standard deviation. *T-test Indiff.* and *t-test Rigidity* show the corresponding t-test statistics. *** indicates *p* < .001*. N =* 998*.*

Importantly, as the final hierarchical regression in Table 10 shows, epistemic vice explains additional variance even when all other measures are included in the regression jointly, from ΔR^2^ = .03 for conspiracist thinking to ΔR^2^ = .09 for susceptibility to Covid-19 misinformation. Table 11 shows detailed results of this hierarchical regression for Covid-19 misinformation, conspiracist thinking, fake news, and overclaiming bias as dependent variables, respectively. For each model there are two steps. Step 1 includes all demographic variables as well as psychological scales. Step 2 adds epistemic indifference and epistemic rigidity. All continuous predictors, as well as the dependent variables, are mean-centered and scaled by one standard deviation. Despite the large number of independent variables, multicollinearity is not a serious concern. Coefficients are fairly stable across outcome variables and when adding or deleting coefficients, and the highest variance inflation factor across these regressions is 3.68, below the threshold of 5 (Hair et al., 2010). Across outcome variables, the EVS shows moderate to large effect sizes. For Covid-19 misinformation and fake news, the coefficients for epistemic vice are the highest coefficients in the regression. This result strongly supports our hypothesis that the EVS explains additional variance with regard to Covid-19 misinformation, over and above the demographic information and the other psychological scales.

**Table 11:** Summary of the hierarchical regression analyses

	Covid-19 Misinfo.		Conspiracy		Fake news		Overclaiming	
	Step 1	Step 2	Step 1	Step 2	Step 1	Step 2	Step 1	Step 2
Epistemic vice: Indifference		0.38 ***		0.20 ***		0.22 ***		0.23 ***
Epistemic vice: Rigidity		0.18 ***		0.12 **		0.25 ***		0.20 ***
Education	0.07 **	0.05 *	0.01	−0.01	0.09 ***	0.06 **	0.09 ***	0.07 ***
Religion	0.13 ***	0.09 ***	0.09 **	0.06 *	0.14 ***	0.10 ***	0.09 ***	0.06 *
Age	−0.09 ***	−0.06 **	−0.09 ***	−0.08 **	−0.06 *	−0.03	−0.11 ***	−0.09 ***
Female	−0.13 **	−0.10 *	−0.15 **	−0.13 **	−0.05	−0.02	−0.15 ***	−0.12 **
Income	−0.08 ***	−0.08 ***	−0.11 ***	−0.11 ***	−0.11 ***	−0.11 ***	−0.08 ***	−0.08 ***
*Political Affiliation*								
Strong Democrat	0.25	0.09	0.26	0.17	0.27	0.16	0.36 *	0.25
Moderate Democrat	0.17	0.01	0.18	0.10	0.21	0.09	0.37 *	0.26
Lean Democrat	0.18	0.02	0.22	0.13	0.23	0.13	0.41 **	0.30 *
Independent	0.14	0.01	0.25	0.18	0.28	0.20	0.31 *	0.22
Lean Republican	0.19	0.06	0.28	0.21	0.41 *	0.31 *	0.48 **	0.38 *
Moderate Republican	0.14	0.03	0.40 *	0.34 *	0.32 *	0.25	0.36 *	0.29 *
Strong Republican	0.37 *	0.14	0.48 **	0.35 *	0.48 **	0.33 *	0.44 **	0.29 *
*Marital Status*								
Married	0.51 *	0.36	0.31	0.23	0.28	0.15	0.19	0.08
Widowed	0.42	0.33	0.22	0.16	0.18	0.09	−0.04	−0.12
Divorced	0.26	0.13	0.06	−0.02	−0.01	−0.12	−0.09	−0.19
Separated	0.48	0.42	0.18	0.14	0.22	0.17	0.15	0.11
Never Married (Dummy)	0.17	0.14	−0.02	−0.04	−0.01	−0.04	−0.18	−0.21
*Ethnicity*								
American Indian or Alaskan Native	0.37	0.00	−0.02	−0.23	0.23	−0.08	0.40	0.12
Asian or Pacific Islander	0.17	0.03	−0.02	−0.10	−0.03	−0.14	0.24	0.15
Black or African American	0.24	0.12	0.05	−0.02	0.09	−0.01	0.28	0.19
Hispanic	0.16	0.05	−0.21	−0.27	0.02	−0.08	0.25	0.16
White / Caucasian	0.01	−0.04	−0.21	−0.24	−0.10	−0.15	0.05	0.01
*Personality*								
Honesty	−0.15 ***	−0.09 ***	−0.14 ***	−0.11 ***	−0.16 ***	−0.10 ***	−0.17 ***	−0.12 ***
Agreeableness	−0.01	−0.02	−0.05	−0.05 *	−0.03	−0.04	−0.05	−0.05 *
Emotionality	0.08 *	0.04	0.11 **	0.09 **	0.11 ***	0.08 **	0.12 ***	0.09 **
Extroversion	−0.01	0.01	0.01	0.02	0.04	0.05 *	0.01	0.02
Conscientiousness	−0.05	−0.01	0.03	0.05	0.01	0.04	0.02	0.05
Intellect	−0.18 ***	−0.08 ***	−0.07 **	−0.02	−0.10 ***	−0.03	−0.16 ***	−0.10 ***
Cognitive reflection	−0.13 ***	−0.08 ***	−0.09 ***	−0.06 **	−0.12 ***	−0.09 ***	−0.16 ***	−0.13 ***
Faith in intuition	0.11 ***	0.01	0.15 ***	0.09 **	0.17 ***	0.06 *	0.15 ***	0.06 *
Open-mindedness	−0.03	0.06 *	−0.01	0.05	0.05	0.12 ***	0.09 **	0.16 ***
Dogmatism	0.09 **	0.04	0.12 ***	0.10 **	0.13 ***	0.08 **	0.17 ***	0.13 ***
Trust in experts	−0.04	−0.04	−0.18 ***	−0.18 ***	−0.16 ***	−0.15 ***	−0.02	−0.02
Need for cognition	0.07 *	0.11 ***	0.08 *	0.10 ***	0.01	0.03	0.04	0.07 **
Need for closure	0.06 *	0.00	0.07 *	0.03	0.06 *	0.01	0.04	−0.01
Self esteem	−0.18 ***	−0.13 ***	−0.24 ***	−0.21 ***	−0.19 ***	−0.15 ***	−0.17 ***	−0.14 ***
Overclaiming: Accuracy	0.15 ***	0.12 ***	0.09 ***	0.07 **	0.04	0.01	0.30 ***	0.28 ***
Constant	−0.59	−0.27	−0.25	−0.07	−0.37	−0.13	−0.46	−0.23
N	973	973	968	968	977	977	977	977
R2	0.60	0.68	0.59	0.62	0.64	0.69	0.65	0.70
**ΔR2**		**0.09**		**0.03**		**0.05**		**0.05**
**ΔF**		130.87***		33.71***		78.38***		70.19***

Four models are tested, with dependent variables Covid-19 misinformation, conspiracy score, fake news score, and overclaiming bias, respectively. Step 1 includes all demographic variables and psychological scales included in the study. Step 2 additionally includes these epistemic indifference and epistemic rigidity. The numbers indicate *β* value. All continuous predictors as well as the dependent variables are mean-centered and scaled by 1 standard deviation. *** *p* < 0.001; ** *p* < 0.01; * *p* < 0.05.

### Discussion

We were able to replicate the factor structure of the epistemic vice scale, with the confirmatory factor analysis showing excellent fit. The epistemic vice scale is correlated to the psychological constructs with the hypothesized sign. However, the epistemic vice scale is not merely redundant with them. The scale predicts a range of relevant outcomes from susceptibility to Covid-19 misinformation and fake news to endorsement of conspiracy theories and overclaiming bias. For each of these outcomes, the epistemic vice scale is a better predictor than any other construct studied. Note that Alfano et al. (2017) investigated associations between their scale measuring intellectual humility and overclaiming bias. The correlations of the epistemic vice scale with overclaiming bias are more than four times higher than what they find. Perhaps the most impressive result is that the epistemic vice scale explains substantial variance over and above all other included scales and demographic variables, for all four outcome measures, and even explains additional variance over all other measures combined.

The EVS captures important components of epistemic vice that have been identified by philosophers and validated by subject matter experts. Using these analyses to inform a broad item pool, two dimensions emerged from our studies: epistemic indifference and epistemic rigidity. Indifference manifests itself in a lack of motivation to find the truth. Rigidity manifests itself in being insensitive to evidence once one’s mind is made up. Further work could test to what extent these two dimensions also underlie individual differences in other epistemic vices that are not covered by the taxonomy we used to generate our item pool, such as excessive malleability.

The validity of these subscales was supported by convergence and divergence with existing scales and the ability of the scale to predict four relevant outcomes. Epistemic vice adds predictive power beyond demographic variables and established psychological scales, suggesting it is not redundant with already-established measures.

A next step for validating the scale would be to reproduce the research outside of North America and in languages other than English. Another next step would be to investigate a broader range of consequences and behaviors. Three of our outcome measures focused on susceptibility to problematic beliefs as the most immediate plausible consequence of epistemic vice: susceptibility to Covid-19 misinformation, to conspiracist thinking, and to fake news. The outcome measure looking at overclaiming bias goes beyond beliefs to behaviors. Future research should investigate a broader range of beliefs as well as behaviors plausibly associated with these beliefs, such as lack of taking important precautions in the case of Covid-19 susceptibility. Yet further work could investigate the development of epistemic vice, as well as interventions that may blunt its effects.

## Supplementary Information

ESM 1 (DOCX 2262 kb)

## Footnotes

1 This study has been pre-registered. Pre-registration available here: https://osf.io/dtgfq?view_only=67dd6ae971354343a84e17fd9ada40ca. Data and code are available here: https://osf.io/yzj3g/?view_only=aeab161c3e3a43f19900584bdfed2ba1

2 Data data and code are available here: https://osf.io/yzj3g/?view_only=aeab161c3e3a43f19900584bdfed2ba1

3 https://www.who.int/emergencies/diseases/novel-coronavirus-2019/advice-for-public/myth-busters; accessed 24 April 2020.

4 Anonymized pre-registration available at url = < https://osf.io/dtgfq?view_only=67dd6ae971354343a84e17fd9ada40ca >.
