# Supplementary material for: The Development and Validation of the Epistemic Vice Scale
Source: Rev Philos Psychol. 2021 Jun 25:1–28. Online ahead of print. doi: 10.1007/s13164-021-00562-5 (PMC8231755; doi:10.1007/s13164-021-00562-5)
Supplement: Supplementary file 1 — (DOCX 2262 kb) [file 13164_2021_562_MOESM1_ESM.docx]

**Online Supplemental Material**

**Study 1**

| Variable | *M* | *SD* | 1 |
| --- | --- | --- | --- |
| 1. Conspiracy | 2.12 | 1.18 |  |
| 2. Apathy1 | 1.43 | 0.64 | .22** |
|  |  |  | [.15, .29] |
| 3. Apathy2 | 1.59 | 0.77 | .18** |
|  |  |  | [.11, .24] |
| 4. Apathy3 | 1.53 | 0.76 | .18** |
|  |  |  | [.11, .25] |
| 5. Apathy4 | 1.41 | 0.66 | .20** |
|  |  |  | [.13, .27] |
| 6. Apathy5 | 1.86 | 1.19 | .48** |
|  |  |  | [.42, .53] |
| 7. Apathy6 | 1.98 | 1.17 | .50** |
|  |  |  | [.45, .55] |
| 8. Apathy7 | 2.00 | 1.24 | .52** |
|  |  |  | [.47, .57] |
| 9. Apathy8 | 1.82 | 1.20 | .50** |
|  |  |  | [.44, .55] |
| 10. Closed1 | 1.90 | 0.84 | .05 |
|  |  |  | [-.02, .12] |
| 11. Closed2 | 2.09 | 0.93 | .20** |
|  |  |  | [.13, .26] |
| 12. Closed3 | 1.55 | 0.77 | .26** |
|  |  |  | [.20, .33] |
| 13. Closed4 | 1.91 | 0.78 | .02 |
|  |  |  | [-.05, .09] |
| 14. Closed5 | 3.21 | 1.07 | .36** |
|  |  |  | [.29, .41] |
| 15. Closed6 | 2.75 | 1.20 | .07* |
|  |  |  | [.00, .14] |
|  |  |  |  |
| 16. Closed7 | 2.57 | 1.26 | .51** |
|  |  |  | [.46, .56] |
| 17. Closed8 | 2.41 | 1.23 | .42** |
|  |  |  | [.36, .47] |
| 18. Sloppiness1 | 1.64 | 0.74 | .11** |
|  |  |  | [.04, .18] |
| 19. Sloppiness2 | 1.69 | 0.74 | .12** |
|  |  |  | [.06, .19] |
| 20. Sloppiness3 | 1.65 | 0.74 | .20** |
|  |  |  | [.13, .26] |
| 21. Sloppiness4 | 1.67 | 0.75 | .08* |
|  |  |  | [.01, .15] |
| 22. Sloppiness5 | 2.21 | 1.26 | .52** |
|  |  |  | [.46, .57] |
| 23. Sloppiness6 | 2.71 | 1.22 | .44** |
|  |  |  | [.38, .49] |
| 24. Sloppiness7 | 2.25 | 1.18 | .51** |
|  |  |  | [.45, .56] |
| 25. Sloppiness8 | 2.83 | 1.25 | .46** |
|  |  |  | [.40, .51] |
| 26. Obstinacy1 | 2.02 | 0.94 | .18** |
|  |  |  | [.11, .24] |
| 27. Obstinacy2 | 1.80 | 0.71 | .12** |
|  |  |  | [.05, .18] |
| 28. Obstinacy3 | 1.68 | 0.72 | .11** |
|  |  |  | [.04, .18] |
| 29. Obstinacy4 | 1.82 | 0.77 | .17** |
|  |  |  | [.10, .23] |
| 30. Obstinacy5 | 3.28 | 1.08 | .33** |
|  |  |  | [.27, .39] |
| 31. Obstinacy6 | 2.80 | 1.21 | .47** |
|  |  |  | [.42, .53] |
| 32. Obstinacy7 | 2.54 | 1.18 | .46** |
|  |  |  | [.40, .51] |
| 33. Obstinacy8 | 2.75 | 1.17 | .44** |
|  |  |  | [.38, .49] |
| 34. Diffidence1 | 1.98 | 0.82 | .12** |
|  |  |  | [.05, .19] |
| 35. Diffidence2 | 2.20 | 0.98 | -.04 |
|  |  |  | [-.10, .03] |
| 36. Diffidence3 | 1.94 | 0.85 | .06 |
|  |  |  | [-.01, .13] |
| 37. Diffidence4 | 2.10 | 0.92 | .05 |
|  |  |  | [-.02, .12] |
| 38. Diffidence5 | 2.26 | 1.19 | .42** |
|  |  |  | [.36, .48] |
| 39. Diffidence6 | 2.63 | 1.25 | .30** |
|  |  |  | [.24, .37] |
| 40. Diffidence7 | 2.54 | 1.21 | .28** |
|  |  |  | [.22, .35] |
| 41. Diffidence8 | 2.52 | 1.21 | .39** |
|  |  |  | [.33, .44] |

**Table S1: Means and standard deviations** **for epistemic vice items and conspiracy score, as well as and correlations between each item and conspiracy score, with confidence intervals**. M and SD are used to represent mean and standard deviation, respectively. Values in square brackets indicate the 95% confidence interval for each correlation. Pairwise Pearson correlations. * indicates p < .05. ** indicates p < .01.

|  | Factor1 | Factor2 | Factor3 | Factor4 |
| --- | --- | --- | --- | --- |
| Apathy5 | 0.75 | -0.02 | 0.05 | 0.02 |
| Apathy6 | 0.74 | 0.04 | 0.05 | 0.01 |
| Apathy7 | 0.72 | 0.08 | 0.00 | 0.03 |
| Apathy8 | 0.87 | -0.05 | 0.00 | 0.01 |
| Closed5 | -0.18 | 0.67 | 0.15 | 0.08 |
| Closed6 | 0.02 | 0.00 | 0.00 | 0.99 |
| Closed7 | 0.15 | 0.56 | 0.05 | 0.00 |
| Closed8 | 0.43 | 0.15 | 0.08 | 0.22 |
| Sloppiness5 | 0.38 | 0.38 | 0.10 | -0.11 |
| Sloppiness6 | 0.17 | 0.53 | 0.00 | 0.00 |
| Sloppiness7 | 0.48 | 0.31 | 0.02 | 0.00 |
| Sloppiness8 | 0.02 | 0.65 | 0.06 | -0.02 |
| Obstinacy5 | -0.05 | 0.55 | -0.06 | 0.07 |
| Obstinacy6 | 0.04 | 0.64 | 0.00 | -0.11 |
| Obstinacy7 | 0.22 | 0.46 | 0.06 | -0.03 |
| Obstinacy8 | 0.03 | 0.73 | -0.05 | 0.09 |
| Diffidence5 | 0.34 | 0.08 | 0.38 | 0.00 |
| Diffidence6 | -0.04 | -0.03 | 0.87 | 0.04 |
| Diffidence7 | 0.17 | 0.14 | 0.33 | 0.11 |
| Diffidence8 | 0.05 | 0.03 | 0.82 | -0.04 |
|  |  |  |  |  |
|  | Factor1 | Factor2 | Factor3 | Factor4 |
| SS loadings | 3.232 | 3.218 | 1.745 | 1.094 |
| Proportion Var | 0.162 | 0.161 | 0.087 | 0.055 |
| Cumulative Var | 0.162 | 0.323 | 0.41 | 0.465 |
|  |  |  |  |  |
| Fit Indices |  |  |  |  |
| RMSR |  |  |  | 0.02 |
| TLI |  |  |  | 0.98 |
| RMSEA |  |  |  | 0.03 |

**Table S2: Four factor solution based on all positively keyed items**


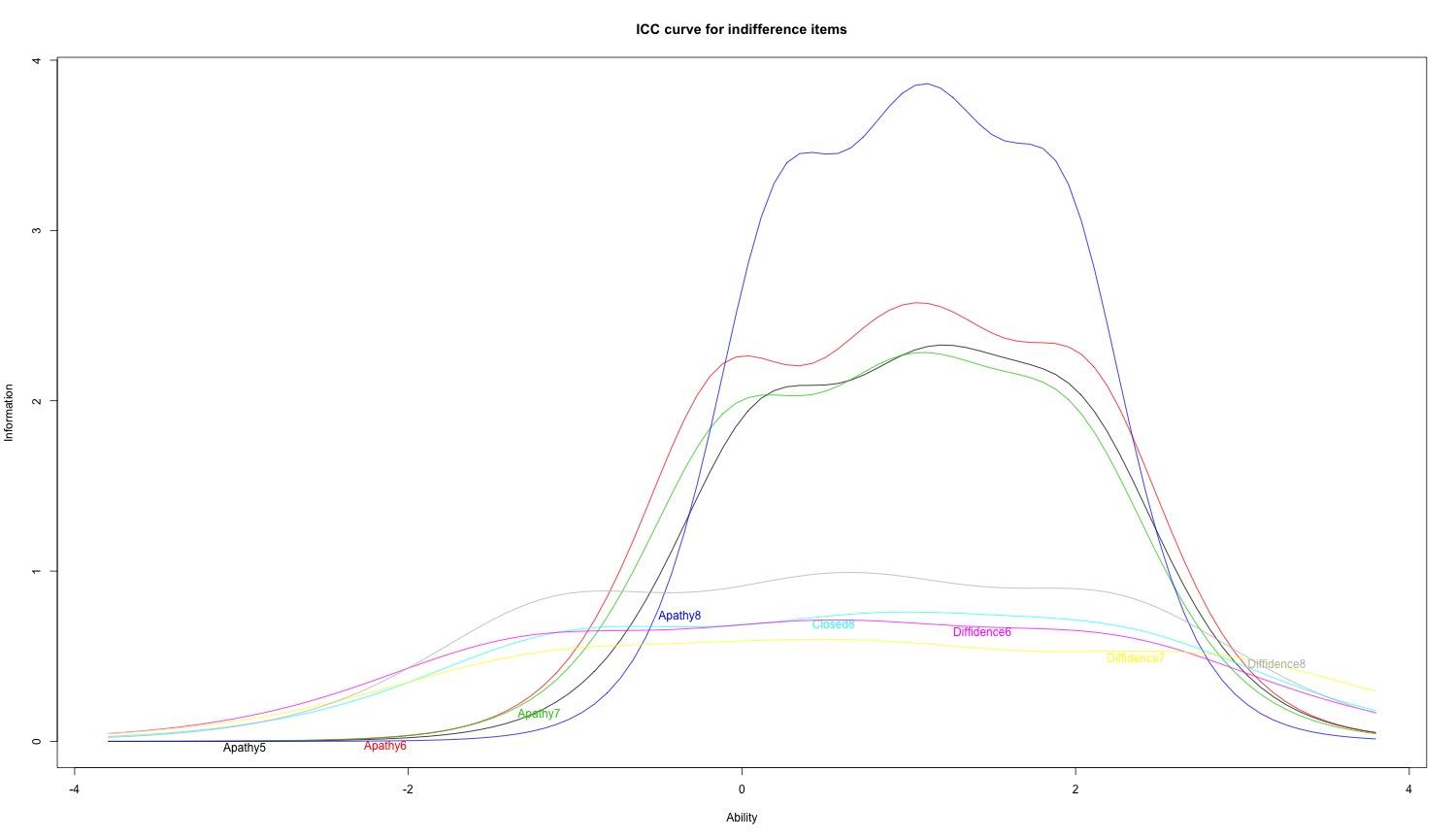


**Figure S1: Item Characteristic curves of indifference items**


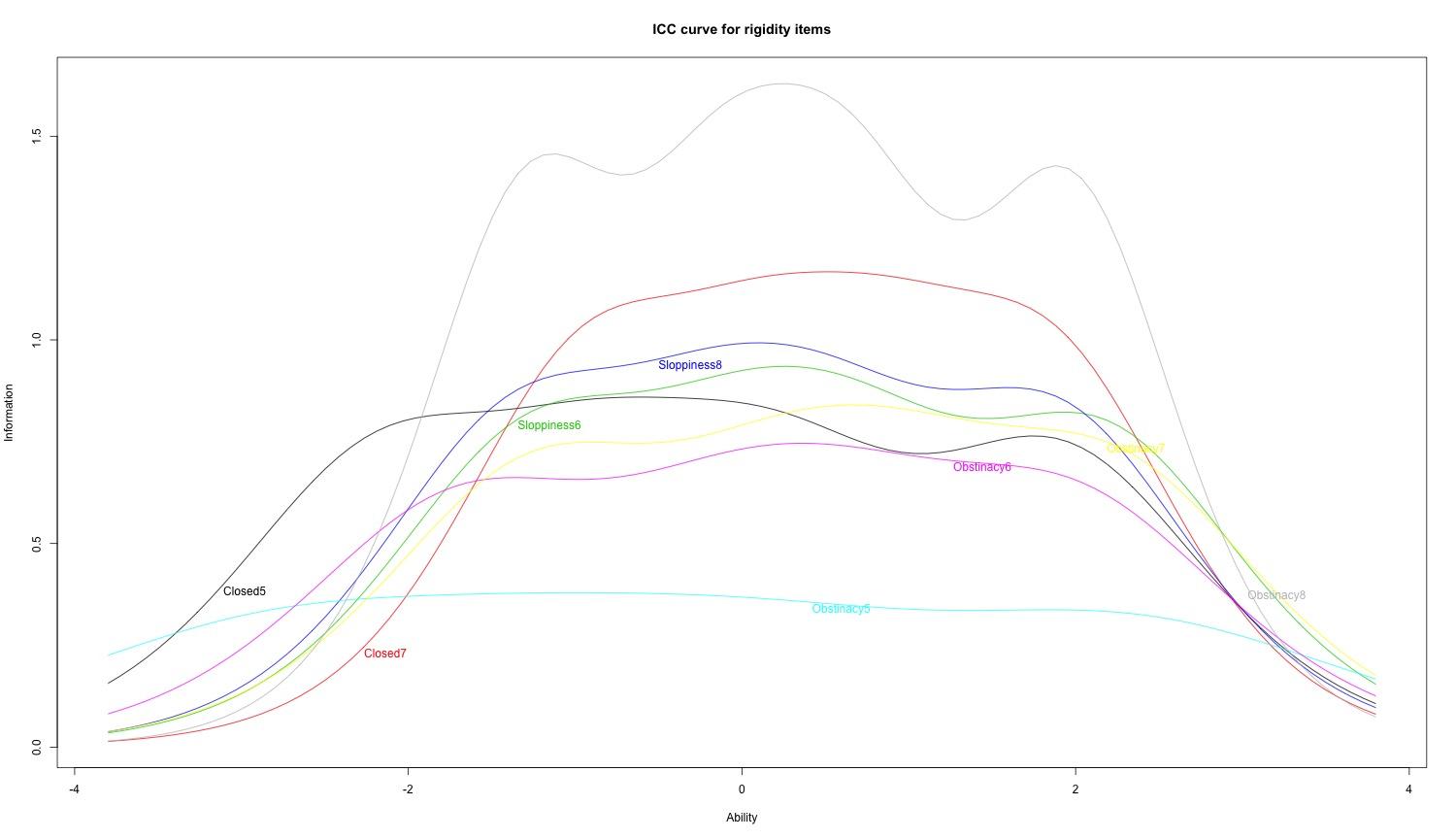


**Figure S2: Item characteristic curves of rigidity items**

**Study 2**


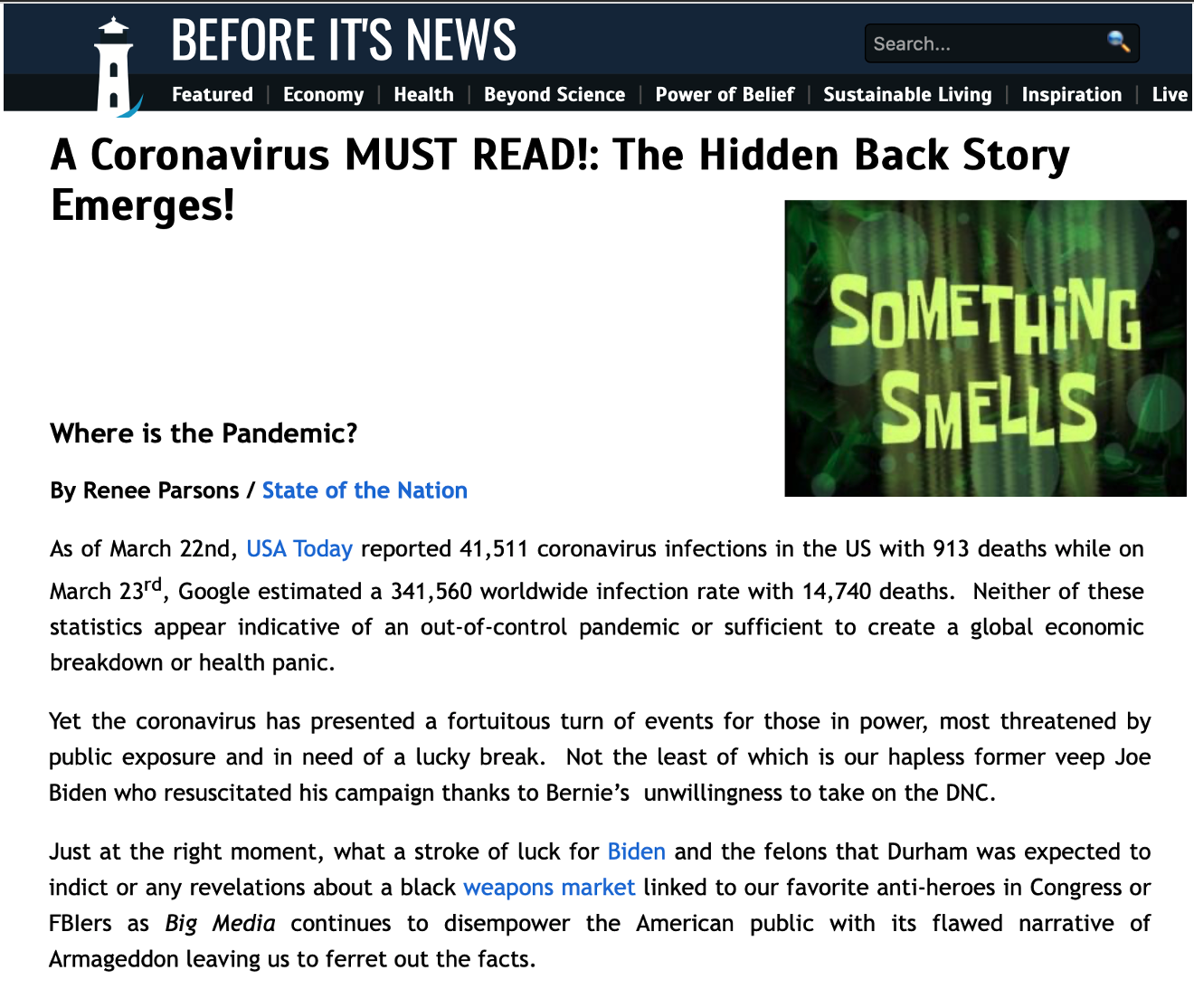


**Figure S3: News item 1 for fake news instrument.**


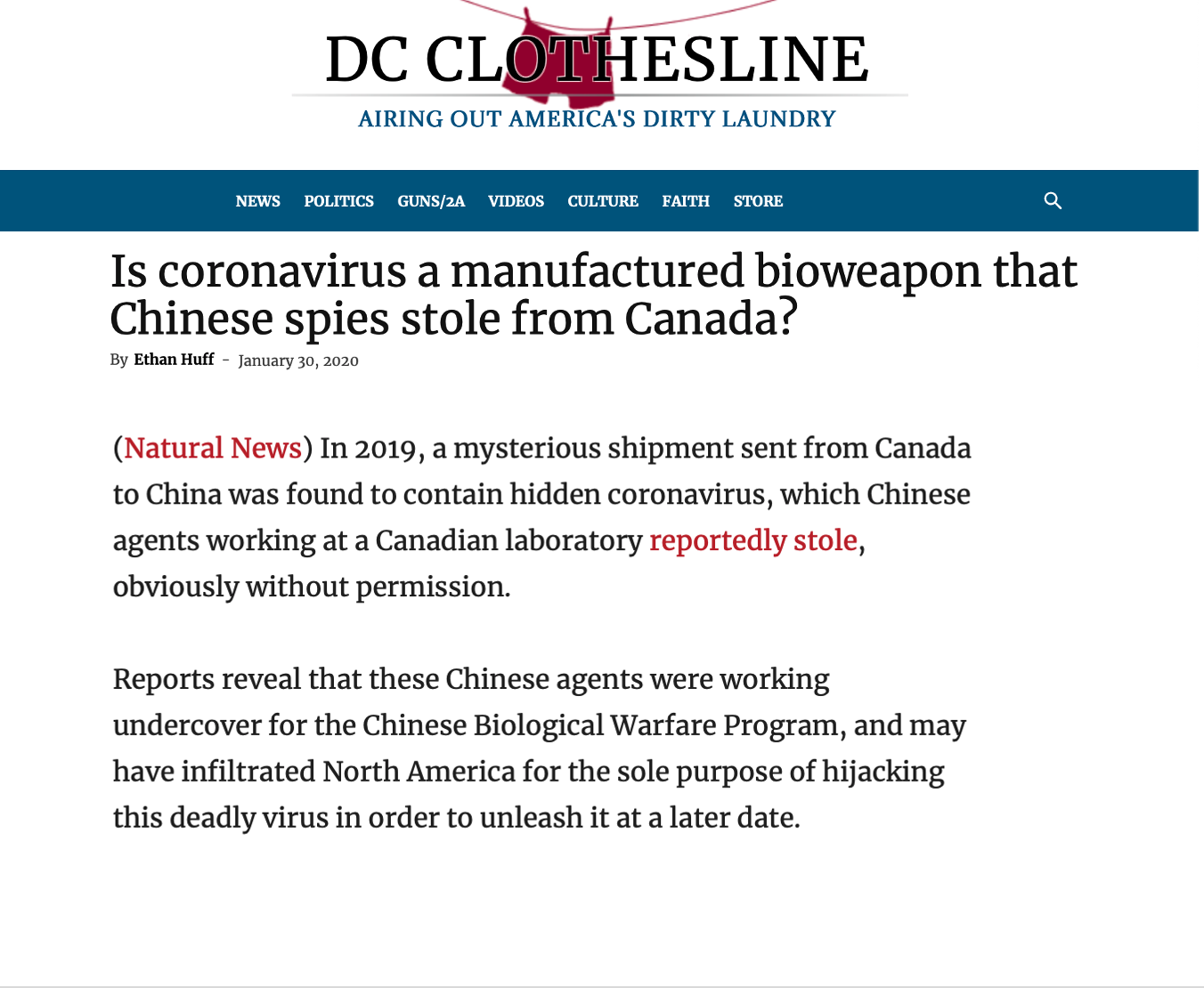


**Figure S4: News item 2 for fake news instrument.**


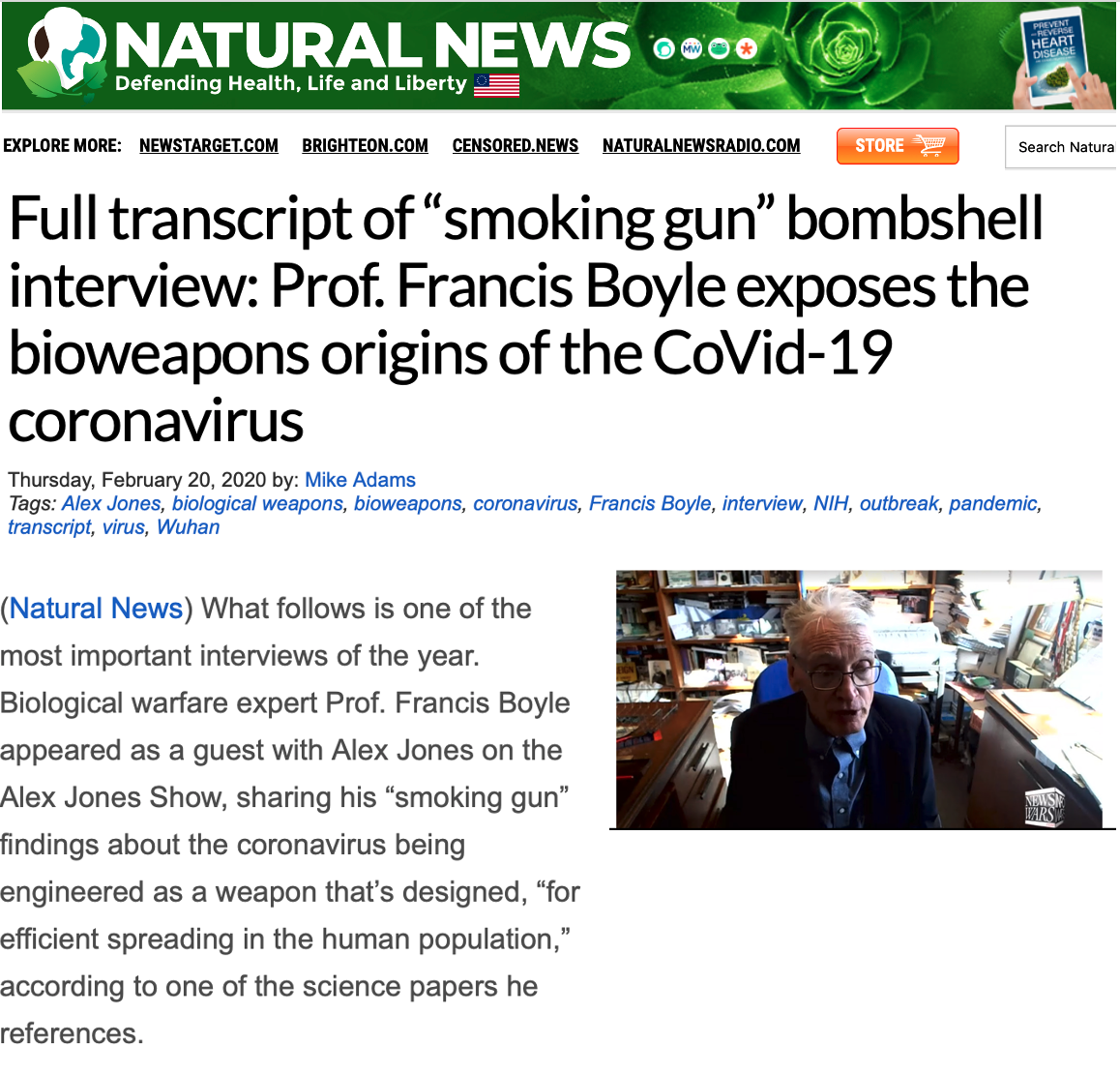
**Figure S5: News item 3 for fake news instrument.**


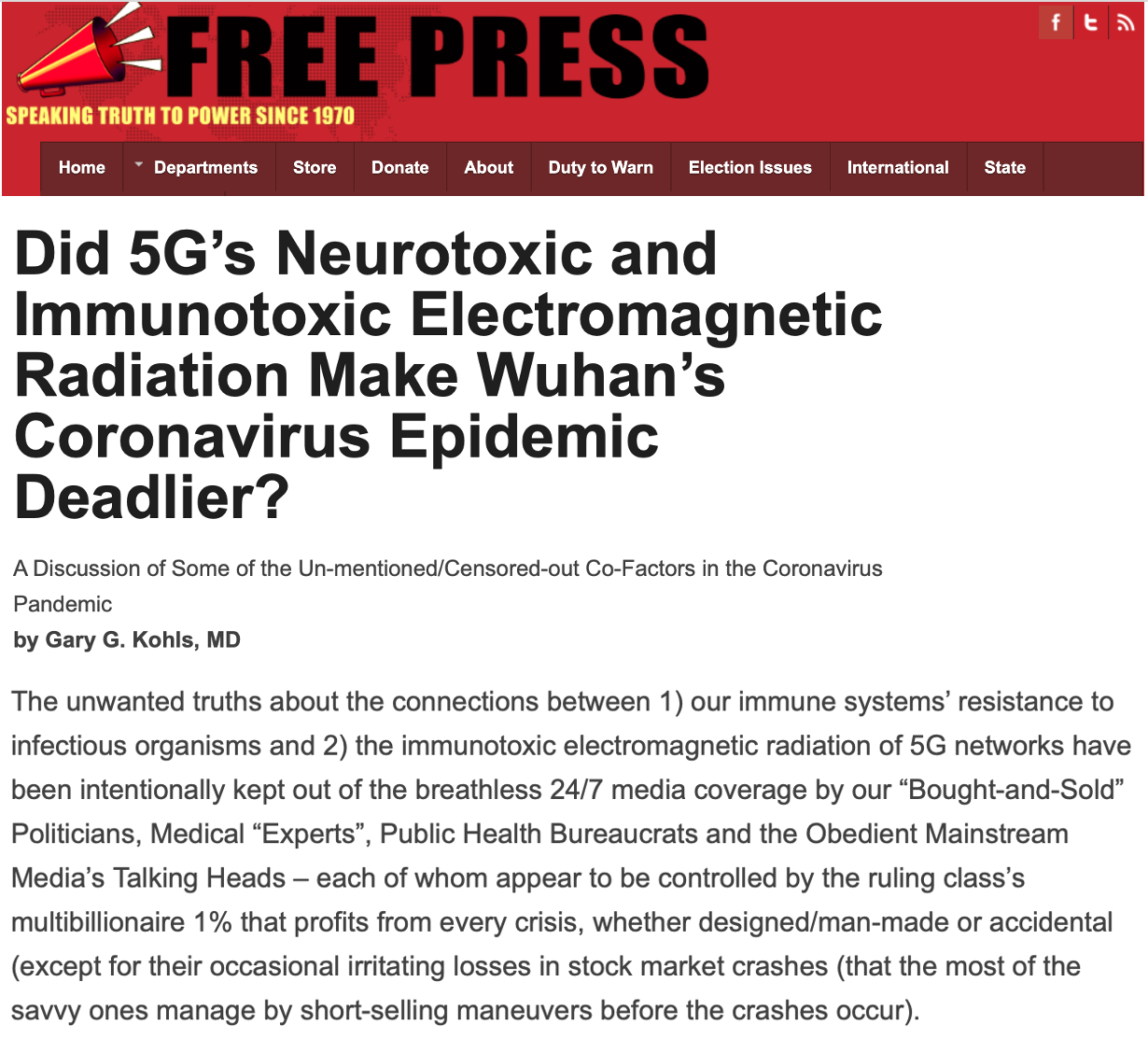


**Figure S6: News item 4 for fake news instrument.**


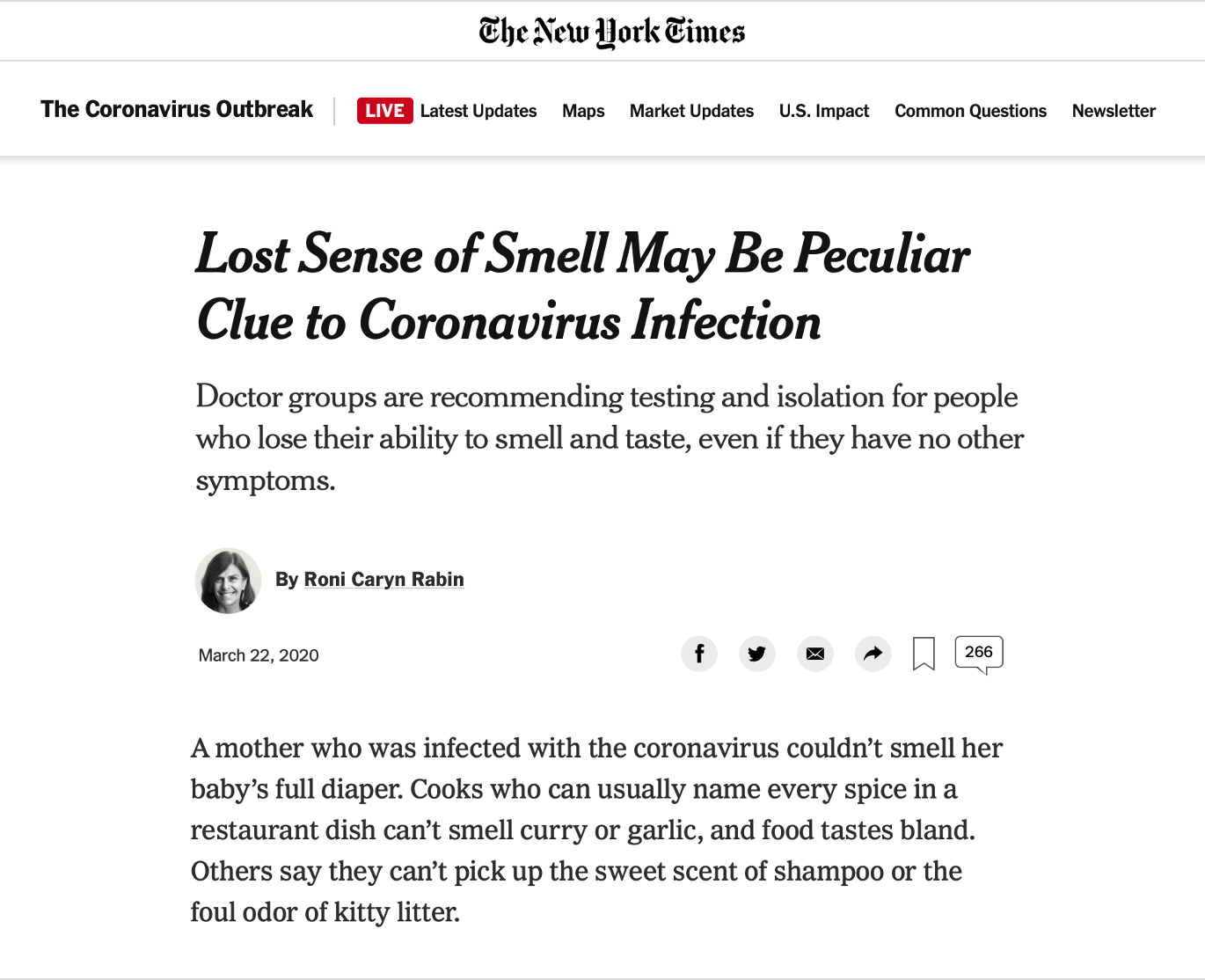


**Figure S7: News item 5 for fake news instrument.** Control item not included in calculating the instrument.

| # | Item | Mean | SD | Skew | Kurtosis |
| --- | --- | --- | --- | --- | --- |
| 1 | It is important to trust experts such as university professors. | 3.70 | 1.02 | -0.77 | 0.18 |
| 2 | The opinion of amateurs is often suppressed, even if these can contribute important content. | 2.66 | 1.12 | 0.35 | -0.73 |
| 3 | Experts provide knowledge that is essential to find good public policies. | 4.05 | 0.81 | -0.84 | 0.99 |
| 4 | Experts are little trustworthy and try to market  their own ideas instead of finding the truth. | 3.25 | 1.22 | -0.17 | -1.04 |
| 5 | Amateurs are more interested in finding the truth because they do not profit from their research. | 2.85 | 1.16 | 0.24 | -0.81 |
| 6 | Experts such as university professors are more objective than amateurs through their methodology. | 3.64 | 1.07 | -0.63 | -0.24 |

**Table S3: Trust in Experts items (adapted from Imhoff et. al 2018).** N=998. Scale = 1-5. SE = 0.04 for items 2, 4 and 5; SE= 0.03 for items 1, 3, and 6.

* = control item not included in calculation of fake news score

| Variable | *M* | *SD* | 1 | 2 | 3 | 4 |
| --- | --- | --- | --- | --- | --- | --- |
| 1. Covid-19 misinformation | 1.88 | 1.08 |  |  |  |  |
| 2. Conspiracy | 2.25 | 1.24 | .78** |  |  |  |
|  |  |  | [.76, .81] |  |  |  |
| 3. Fake News | 2.34 | 1.22 | .79** | .82** |  |  |
|  |  |  | [.77, .81] | [.80, .84] |  |  |
| 4. Overclaiming Bias | 2.17 | 1.10 | .79** | .71** | .76** |  |
|  |  |  | [.76, .81] | [.68, .74] | [.73, .78] |  |
| 5. Epistemic Vice | 2.49 | 0.90 | .76** | .68** | .75** | .71** |
|  |  |  | [.73, .79] | [.65, .71] | [.72, .77] | [.68, .74] |
| 6. Indifference | 1.97 | 1.06 | .72** | .60** | .64** | .62** |
|  |  |  | [.69, .75] | [.55, .63] | [.60, .67] | [.58, .66] |
| 7. Rigidity | 2.83 | 0.92 | .68** | .65** | .72** | .67** |
|  |  |  | [.64, .71] | [.61, .68] | [.69, .75] | [.64, .71] |
| 8. Dogmatism | 2.49 | 0.66 | .47** | .52** | .56** | .49** |
|  |  |  | [.42, .52] | [.48, .57] | [.51, .60] | [.44, .53] |
| 9. Faith in Intuition | 3.32 | 0.81 | .45** | .51** | .55** | .50** |
|  |  |  | [.40, .50] | [.46, .56] | [.51, .60] | [.45, .55] |
| 10. Personality:Honesty | 3.61 | 0.77 | -.43** | -.40** | -.39** | -.43** |
|  |  |  | [-.48, -.38] | [-.45, -.34] | [-.44, -.34] | [-.48, -.38] |
| 11. Importance of Religion | 2.65 | 1.49 | .42** | .43** | .50** | .41** |
|  |  |  | [.37, .47] | [.37, .48] | [.46, .55] | [.36, .46] |
| 12. Openmindedness | 3.74 | 0.72 | -.42** | -.42** | -.39** | -.34** |
|  |  |  | [-.47, -.37] | [-.47, -.36] | [-.45, -.34] | [-.39, -.28] |
| 13. Cognitive Reflection | 0.49 | 0.34 | -.39** | -.39** | -.44** | -.41** |
|  |  |  | [-.44, -.33] | [-.44, -.33] | [-.49, -.39] | [-.46, -.36] |
| 14. Personality:Intellect | 3.55 | 0.65 | -.38** | -.30** | -.33** | -.29** |
|  |  |  | [-.43, -.32] | [-.36, -.25] | [-.38, -.27] | [-.35, -.23] |
| 15. Self-Esteem | 3.67 | 0.79 | -.35** | -.30** | -.26** | -.25** |
|  |  |  | [-.40, -.29] | [-.36, -.24] | [-.31, -.20] | [-.31, -.19] |
| 16. Need for Closure | 3.43 | 0.71 | .30** | .33** | .33** | .27** |
|  |  |  | [.25, .36] | [.28, .39] | [.28, .39] | [.21, .33] |
| 17. Trust in Experts | 3.36 | 0.71 | -.30** | -.45** | -.45** | -.27** |
|  |  |  | [-.36, -.24] | [-.50, -.40] | [-.49, -.40] | [-.32, -.21] |
| 18. Personality:Conscientiousn. | 3.76 | 0.82 | -.27** | -.17** | -.15** | -.18** |
|  |  |  | [-.32, -.21] | [-.23, -.11] | [-.21, -.09] | [-.24, -.12] |
| 19. Personality:Agreeableness | 3.46 | 0.79 | -.26** | -.28** | -.24** | -.23** |
|  |  |  | [-.32, -.20] | [-.33, -.22] | [-.30, -.18] | [-.29, -.17] |
| 20. Need for Cognition | 3.36 | 0.81 | -.25** | -.26** | -.30** | -.18** |
|  |  |  | [-.30, -.19] | [-.32, -.20] | [-.36, -.24] | [-.24, -.12] |
| 21. Education | 4.87 | 0.96 | .23** | .12** | .18** | .28** |
|  |  |  | [.17, .29] | [.06, .18] | [.12, .24] | [.22, .34] |
| 22. Political Affiliation | 3.69 | 2.26 | .19** | .29** | .31** | .18** |
|  |  |  | [.12, .25] | [.23, .35] | [.24, .37] | [.11, .25] |
| 23. Personality:Emotionality | 3.37 | 0.86 | -.19** | -.14** | -.11** | -.10** |
|  |  |  | [-.25, -.13] | [-.21, -.08] | [-.17, -.05] | [-.16, -.03] |
| 24. Overclaiming Accuracy | 3.39 | 0.75 | .21** | .13** | .08* | .34** |
|  |  |  | [.15, .27] | [.07, .19] | [.02, .14] | [.28, .39] |
| 25. Personality:Extroversion | 3.40 | 0.76 | -.17** | -.13** | -.08* | -.08** |
|  |  |  | [-.23, -.11] | [-.19, -.07] | [-.14, -.01] | [-.14, -.02] |
| 26. Age | 39.39 | 12.36 | -.15** | -.13** | -.12** | -.16** |
|  |  |  | [-.21, -.09] | [-.19, -.06] | [-.18, -.05] | [-.22, -.10] |
| 27. Income | 64574.47 | 38988.49 | -.10** | -.13** | -.12** | -.05 |
|  |  |  | [-.16, -.04] | [-.19, -.06] | [-.18, -.05] | [-.11, .01] |
| 28. Female | 0.37 | 0.48 | -.08* | -.08** | -.02 | -.09** |
|  |  |  | [-.14, -.02] | [-.15, -.02] | [-.09, .04] | [-.15, -.03] |

**Table S4: Means, standard deviations, and correlations with confidence intervals**

The table shows correlations between covariates in percentages (pairwise Pearson correlations). *M* and *SD* are used to represent mean and standard deviation, respectively. Values in square brackets indicate the 95% confidence interval for each correlation. * indicates *p* < .05. ** indicates *p* < .01.
